# Radiation Dose Reduction and Image Quality Improvement of UHR CT of the Neck by Novel Deep-learning Image Reconstruction

**DOI:** 10.1007/s00062-025-01532-5

**Published:** 2025-06-30

**Authors:** Dominique Alya Messerle, Nils F. Grauhan, Laura Leukert, Ann-Kathrin Dapper, Roman H. Paul, Andrea Kronfeld, Bilal Al-Nawas, Maximilian Krüger, Marc A. Brockmann, Ahmed E. Othman, Sebastian Altmann

**Affiliations:** 1 Department of Neuroradiology, University Medical Center Mainz, Johannes Gutenberg University, Langenbeckstr. 1, 55131 Mainz, Germany; 2 Institute of Medical Biostatistics, Epidemiology and Informatics (IMBEI), University Medical Center Mainz, Johannes Gutenberg University, Rhabanusstr. 3/Tower A, 55118 Mainz, Germany; 3 Department of oral and maxillofacial surgery, University Medical Center Mainz, Johannes Gutenberg University, Langenbeckst. 1, 55131 Mainz, Germany

**Keywords:** UHR-CT, Deep learning, Image quality, Head and neck, Radiation dose

## Abstract

**Purpose:**

We evaluated a dedicated dose-reduced UHR-CT for head and neck imaging, combined with a novel deep learning reconstruction algorithm to assess its impact on image quality and radiation exposure.

**Methods:**

Retrospective analysis of ninety-eight consecutive patients examined using a new body weight-adapted protocol. Images were reconstructed using adaptive iterative dose reduction and advanced intelligent Clear-IQ engine with an already established (DL-1) and a newly implemented reconstruction algorithm (DL-2). Additional thirty patients were scanned without body-weight-adapted dose reduction (DL-1-SD). Three readers evaluated subjective image quality regarding image quality and assessment of several anatomic regions. For objective image quality, signal-to-noise ratio and contrast-to-noise ratio were calculated for temporalis and masseteric muscle and the floor of the mouth. Radiation dose was evaluated by comparing the computed tomography dose index (CTDIvol) values.

**Results:**

Deep learning-based reconstruction algorithms significantly improved subjective image quality (diagnostic acceptability: DL‑1 vs AIDR OR of 25.16 [6.30;38.85], *p* < 0.001 and DL‑2 vs AIDR 720.15 [410.14;> 999.99], *p* < 0.001). Although higher doses (DL-1-SD) resulted in significantly enhanced image quality, DL‑2 demonstrated significant superiority over all other techniques across all defined parameters (*p* < 0.001). Similar results were demonstrated for objective image quality, e.g. image noise (DL‑1 vs AIDR OR of 19.0 [11.56;31.24], *p* < 0.001 and DL‑2 vs AIDR > 999.9 [825.81;> 999.99], *p* < 0.001). Using weight-adapted kV reduction, very low radiation doses could be achieved (CTDIvol: 7.4 ± 4.2 mGy).

**Conclusion:**

AI-based reconstruction algorithms in ultra-high resolution head and neck imaging provide excellent image quality while achieving very low radiation exposure.

**Supplementary Information:**

The online version of this article (10.1007/s00062-025-01532-5) contains supplementary material, which is available to authorized users.

## Introduction

Computed tomography (CT) is an essential part of emergency radiology and neuroradiology and is an integral part of everyday clinical practice [1, 2]. While magnetic resonance imaging (MRI) offers superior soft tissue contrast and is preferred, particularly for younger patients due to its lack of radiation, certain anatomical regions are easier to assess on CT than on MRI [3, 4]. In addition, significantly higher resolutions and lower slice-thickness can be achieved using Ultra-high-resolution CT (UHR-CT) [5]. Thus, CT remains an important tool for the initial staging and monitoring of head and neck cancer patients, not only due to economic considerations but also to the advantage of rapid image acquisition and, thus, robustness against movement artifacts. As many head and neck cancer patients are older and may have pre-existing conditions or impaired health, it is essential to reduce examination time to a minimum in order to minimize motion artifacts and achieve high-quality diagnostic images.

Technological advancements through a combination of deep-learning-based image reconstruction with advanced intelligent Clear-IQ Engine (AiCE) and hardware improvements have continuously pushed forward image quality and thus increased diagnostic confidence [6–9]. Thereby, UHR-CT enables a very thin slice thickness of 0.25 mm and enhanced visualization of minute structures, e.g. in the temporal bone. However heightened resolutions resulted in higher image noise compared to normal Multidetector-CT [10, 11]. This is where the possibility of noise reduction through deep-learning reconstruction becomes important [12]. The image quality through the deep learning-based image reconstruction of UHR-CT images is already outstanding. Yet, recent developments in image reconstruction show further potential for improving image quality, thereby unlocking new possibilities. CT is still most often the primary imaging modality, as it is significantly more available than MRI, especially in case of emergency examinations and during on-call hours. Furthermore, when there is clinical suspicion of possible airway obstruction if it is not promptly addressed, CT scans are also frequently used for younger patients. As medical radiation exposure steadily increases, it becomes increasingly important to balance achieving acceptable image quality and minimizing radiation exposure, which aligns with the ALARA (As Low As Reasonably Achievable) principle [13].

We hypothesized that combining a recently implemented deep learning-based reconstruction algorithm with tube current modulation and body weight-adapted tube voltage would enable significantly reduced radiation exposure for head and neck imaging while at least maintaining diagnostic image quality.

## Material and Methods

This retrospective study was approved by the Ethics Committee of the Rhineland-Palatinate Chamber of Physicians, and informed consent was waived along with the ethical approval number 2021-15948_3 and the approval date 20 November 2024. The study was conducted in accordance with the revised Helsinki Declaration from 2013. Full control of participant data was maintained by the authors, who are not employees of Canon Medical.

### Patient Cohort

**Fig. 1:**
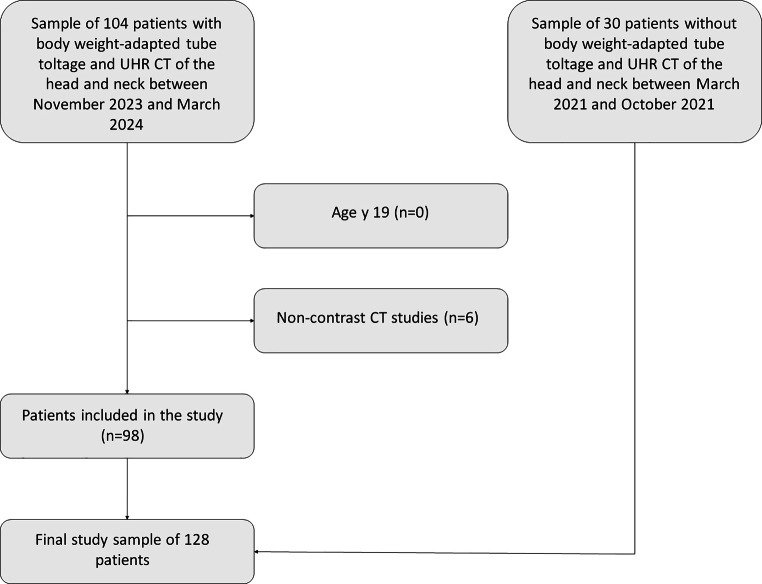
Flow chart of the inclusion/exclusion process

Between November 2023 and March 2024, 98 consecutive patients underwent contrast enhanced UHR-CT of the head and neck examined using tube current modulation and body weight-adapted tube voltage (Patient characteristics are defined in Table 1). Furthermore, another 30 patients with conventional contrasted-enhanced UHR CT of the head and neck, without weight-adapted dose modulation were examined between March 2021 and October 2021. These patients were matched regarding patient age and weight (Supplementary Table S1). The exclusion criteria were (i) age younger than 18 years, (ii) non-contrast CT studies (including extravasation). The inclusion/exclusion process is presented in Fig. 1.

**Table 1:** Patient characteristics

Characteristics	Value (*n* = 98)
Age (years)*	67.8 ± 14.5 (18–92)
Median age	70
**Sex**	
Male	51 (52%)
Female	47 (48%)
**Weight (kg)**	72.3 ± 15.1 (42–119)
**Pathologic findings**	
*Squamous cell carcinoma*	67 (66.3%)
Lower jaw	21 (31.3%)
Tongue	16 (23.9%)
Floor of the mouth	12 (17.9%)
Cheek	8 (11.9%)
Upper jaw	6 (9.2%)
Lips	2 (2.9%)
Palate	2 (2.9%)
**Inflammatory processes (abscess, salivary gland inflammation)**	11 (11.2%)
**Melanoma**	2 (2.0%)
**Osteosarcoma**	1 (1.0%)
**Carcinoma of the hypopharynx**	1 (1.0%)
**Myxofibroma of the upper jaw**	1 (1.0%)
**Spinalioma of the scalp**	1 (1.0%)
**Papilloma of the tongue**	1 (1.0%)
**Others**	13 (13.3%)

* Data are mean ±1 SD, with ranges in parentheses

### Image Acquisition and Reconstruction

UHR-CT images were obtained using an Aquilion Precision scanner (Canon Medical Systems), featuring a focal spot size of 0.4 mm × 0.5 mm (smallest), detector elements measuring 0.25 mm × 0.25 mm, a slice thickness of 0.25 mm, a reconstruction matrix of 1024 × 1024, and beam collimation of 0.25 mm × 160 rows with 1792 channels. The scans were performed using tube current modulation and body weight-adapted tube voltage (80 kV for patients ≤ 70 kg, 100 kV for patients > 70 and ≤ 80 kg, and 120 kV for patients > 80 kg). The images were reconstructed using AIDR (adaptive iterative dose reduction) and AiCE (advanced intelligent Clear-IQ engine), thereby using the former DL‑1 (established AiCE-Body reconstruction) as well as a second recently developed reconstruction algorithm named AiCE-CTA (DL-2). Furthermore, additional 30 consecutive patients were examined without body weight-adapted tube voltage (120 kV), resulting in higher radiation doses (DL-1-SD). The four different imaging techniques are arranged in Supplementary Fig. 1. The AiCE algorithms were trained using high-quality model-based iterative reconstruction (MBIR) images as gold standard targets. The deep convolutional neural network learned to transform lower-quality input images, acquired at 12.5%, 25%, 50%, and 75% of the reference dose, into high-quality outputs by minimizing the error between its predictions and the MBIR target images. This input-forward, error-backpropagation approach was repeated iteratively until optimal performance is achieved. AiCE operates in both the raw and image domains, enabling highly effective noise reduction and signal preservation. While MBIR offers superior image quality, its long reconstruction time makes it unsuitable for routine clinical use. However, it serves as an ideal target for supervised learning during the AiCE training process [14, 15].

The key difference between AiCE Body and AiCE Brain CTA lies in the type of training data:

- AiCE Body (DL-1) was trained using a wide variety of clinical datasets from body regions such as the abdomen, reconstructed at multiple dose levels and scan conditions.
- AiCE Brain CTA (DL-2) followed the same supervised learning pipeline but was trained with dedicated clinical neurovascular CTA datasets, enabling the model to better preserve fine vascular structures critical in brain and neck imaging.

To ensure robustness in clinical application, AiCE was also trained using diverse reconstruction conditions, including different reconstruction fields-of-view (FOVs). This allows the network to adapt to application-dependent variations and maintain consistent image quality.

Contrast injection was conducted through a high-pressure syringe system for advanced clinical CT imaging procedures (Accutron CT‑D; Medtron; Saarbrücken, Germany), using a nonionic contrast agent (iopromide, Ultravist-370; Bayer Healthcare, Germany) via an 18 G peripheral venous catheter placed in the cubital vein. A total of 110 ml Ultravist 370 was injected. Thereby at first, 65 ml of the contrast agent was injected at a flow rate of 1.5 ml per second (injection time 44 s), immediately followed by a 25 ml saline bolus (flow rate 2.0 ml/s; injection time 12s) and a second 10 ml saline bolus (flow rate 0.1 ml/injection time 100 s). Thereafter a second contrast bolus of 50 ml was administered (flow rate 5 ml/injection time 17s) CT-Scan automatically started 180 s after start of the Injection.

### Subjective Image Evaluation

The subjective quality of the images was assessed by two radiology residents (one year and two years of experience in head and neck imaging) and a board-certified neuroradiologist (eight years of experience in head and neck imaging). All reviewers were briefed in applying a 5-point Likert scale (Supplementary Table S2). The 5‑point Likert scale was consistently applied across all patients and categories. The raters evaluated image noise, sharpness, occurrence of artifacts, and overall diagnostic acceptability. Additionally, they assessed the visibility of specific anatomic regions, including nasopharyngeal space, oropharyngeal space, hypopharyngeal space, oral cavity, the floor of the mouth, salivary glands, lymph nodes levels and the pterygopalatine fossa. To minimize recall bias, the images were randomized and assessed after a washout period of two weeks. Any participant- or sequence-identifying markers were removed.

### Objective Image Evaluation

To assess the objective image quality, one or more regions of interest (ROI) were drawn for the quantitative evaluation containing one or two tissue types. For each tissue type, the signal-to-noise ratio (SNR) and the entropy of the grey values were determined. If two tissue types were present in one ROI, the contrast-noise-ratio (CNR) and the sharpness of the transition from one tissue to the other were calculated.

The signal intensity was defined as the modal value derived from the histogram with a bin-width of 20 for every tissue type. To measure the noise, we created a lowpass filtered version of the original image and then subtracted it from the original image to isolate the high predominant noise-related frequencies.

**Fig. 2:**
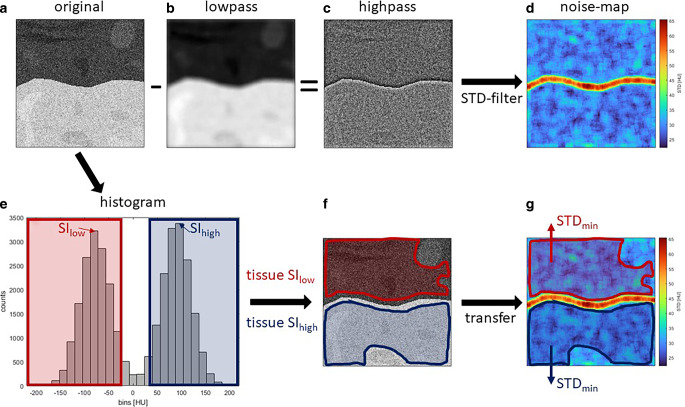
SNR- and CNR-calculation for two tissues: From the chosen ROI (**a**), a highpass filtered copy (**c**) was derived by subtracting a lowpass filtered version (**b**). A noise map (**d**) was derived by filtering the highpass copy (**c**) using a STD filter. The two most frequent SI values in the lower and the higher SI ranges were determined histogram-based (**e**). Two regions, one with low SI and one with higher SI, were derived (**f**). The regions were transferred to the noise map, and the minimum STD for each region was determined (**g**). SNR and CNR were calculated from the SI and STD following Eq. 1 and 2; *ROI* region of interest, *STD* standard deviation, *SI* signal intensity, *SNR* signal to noise Ratio, *CNR* contrast to noise ratio

Noise STDmin of the ROI was calculated as the mean of the five lowest standard deviation values of the noise image derived from circular regions with a diameter of 12 pixels. SNR was calculated as the quotient of SI and STDmin for each ROI and each tissue type (Eq. 1). If a ROI contained two types of tissues, CNR was calculated as the difference of both SI’s divided by the mean of both STDmin (Eq. 2) as, demonstrated in Fig. 2.

1 $${SNR}=\frac{{SI}}{{STD}_{{\min }}}$$

2 $${CNR}=\frac{{\Delta }{SI}}{\overline{{STD}}_{{\min }}}$$

### Radiation Dose

We evaluated the computed tomography dose index (CTDIvol) to estimate the radiation dose. Furthermore, kV values were collected. For comparability between the two groups, further dose calculation was done with a median scan length of 25 cm, similar to already published data [9, 16]. The effective dose values were calculated by multiplying the normalized dose length product with the International Commission on Radiological Protection conversion factor for head and neck CT (k = 0.0058) [17].

### Statistical Analysis

Statistical analysis was performed using SAS 9.4 (SAS Institute, Cary NC). Continuous variables were reported with mean and standard deviation, and ordinal variables were reported using the median and the interquartile range. Gwet’s AC_2_, which is appropriate in an ordinal setting with multiple raters, was performed to assess interrater agreement [18]. AC_2_ values of less than 0.40 were considered a poor agreement, 0.40 to 0.75 was considered a good agreement, while values above 0.75 were considered an excellent agreement, following the benchmark suggestion by Fleiss [19].

For comparison of ordinal data, proportional odds generalized estimating equations (GEE) models were applied. This approach is well-suited for ordinal dependent variables, as it models the cumulative probabilities of the outcome categories while accounting for the inherent order in the data. Patients and readers were included as random effects to properly account for within-subject and within-reader correlation and thus obtaining correct standard errors. To quantify how the employed acquisition methods compare against each other, odds ratios with corresponding 95%-confidence intervals were calculated from the model estimates. Wald tests derived from the GEE model delivered the corresponding *p*-values.

Continuous data was compared with similar considerations using mixed models employing the patients as random effects. *P*-values of less than 0.05 were considered statistically significant.

## Results

### Patient Cohort

The final study sample consisted of 98 patients (51 men and 47 women), with a mean age of 67.8 years (age ranging between 18–92 years). Patient characteristics are demonstrated in Table 1 and Supplementary Table S1.

### Subjective Image Quality

**Fig. 3:**
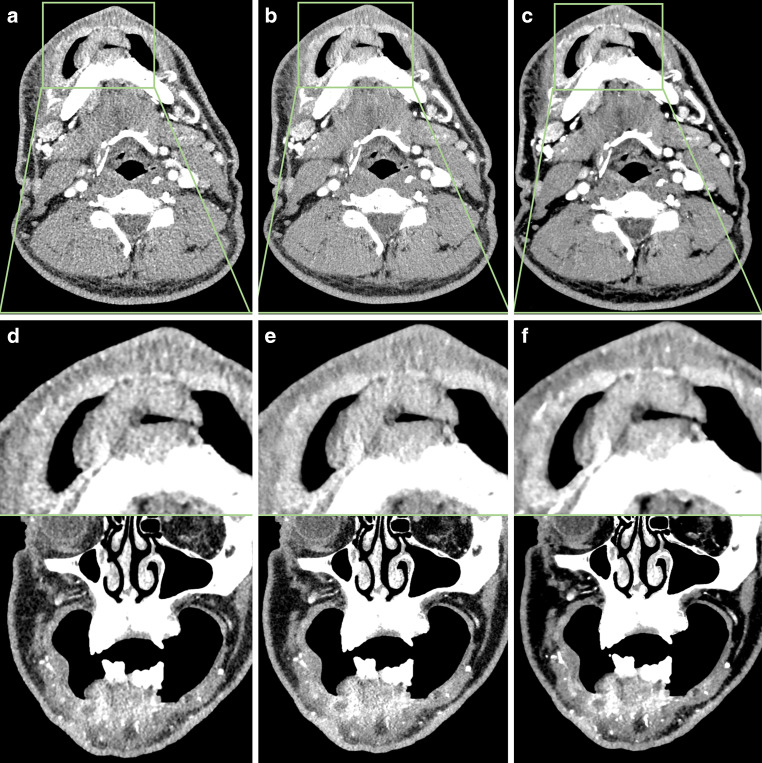
61-year-old male weighing 70 kg with a polypoid, midline squamous cell carcinoma of the lower jaw. Note the increased soft tissue differentiation and the sharp vessel differentiation, possible with the novel deep-learning reconstruction. 0.25 mm reconstruction **a** axial AIDR, **b** axial DL‑1, **c** axial DL‑2, **d** coronal AIDR, **e** coronal DL‑1, **f** coronal DL‑2; *AIDR* Adaptive Iterative Dose Reduction; *DL‑1* Deep-learning Reconstruction; *DL‑2* Novel Deep-learning Reconstruction

**Fig. 4:**
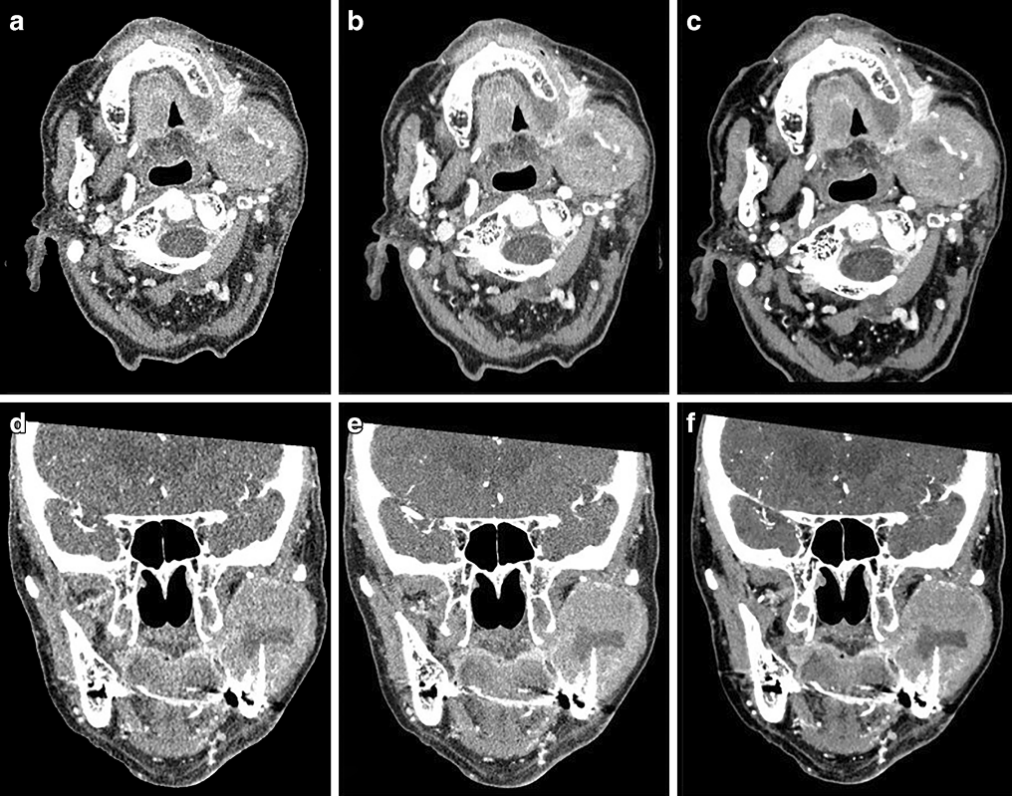
84-year-old female weighing 65 kg with extensive squamous cell carcinoma of the left mandible. Scans were acquired with a CTDI of 4.3 mGy; 0.25 mm reconstruction **a** axial AIDR, **b** axial DL‑1, **c** axial DL‑2, **d** coronal AIDR, **e** coronal DL‑1, **f** coronal DL‑2; *AIDR* Adaptive Iterative Dose Reduction; *DL‑1* Deep-learning Reconstruction; *DL‑2* Novel Deep-learning Reconstruction

**Fig. 5:**
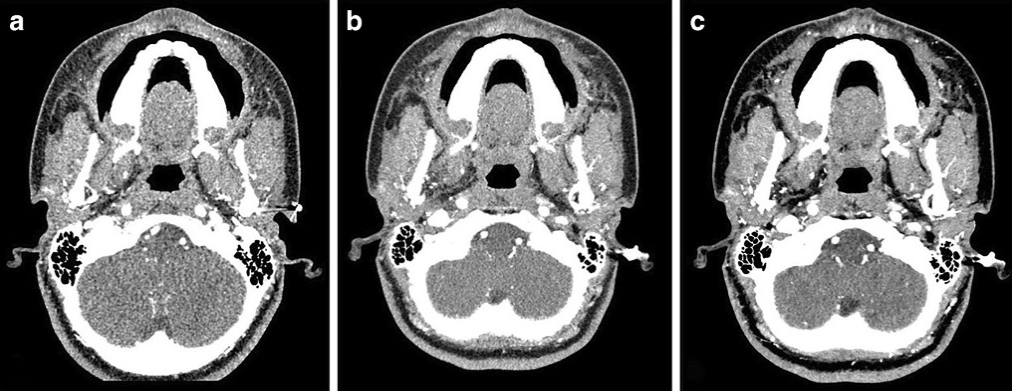
18-year-old female with 62 kg and a phlegmoneous inflammation after wisdom tooth extraction. Scans were acquired with a CTDI of 5 mGy; 0.25 mm reconstruction; **a** axial AIDR, **b** axial DL‑1, **c** axial DL‑2; *AIDR* Adaptive Iterative Dose Reduction; *DL‑1* Deep-learning Reconstruction; *DL‑2* Novel Deep-learning Reconstruction

Both deep-learning-based reconstruction techniques resulted in significantly better image quality (Supplementary Table S3). The DL‑1 technique outperformed normal iterative reconstruction, confirming previous expectations. Considering DL-1-SD, we could demonstrate that higher dose values resulted in significantly better image quality (Tables 2 and 3 and Supplementary Table S4). However, DL‑2 reconstruction algorithm, combined with tube current modulation and body weight-adapted tube voltage, demonstrated significant superiority over the other techniques across all defined parameters (Fig. 3) e.g. diagnostic acceptability (DL‑1 vs AIDR with OR of 25.16 [6.30; 38.85], *p* < 0.001 and DL‑2 vs AIDR 720.15 [410.14;> 999.99], *p* < 0.001) and image noise (DL‑1 vs AIDR with OR of 19.0 [11.56; 31.24], *p* < 0.001 and DL‑2 vs AIDR > 999.9 [825.81;> 999.99], *p* < 0.001). Overall, DL‑2 resulted in excellent image quality in most cases (76.2% excellent diagnostic acceptability for DL‑2 vs 13% for DL‑1 and DL-1-SD and only 0.3% for plain AIDR; Supplementary Table S3). Figures 4 and 5 show examples of a squamous cell carcinoma of the left mandible and a phlegmoneous inflammation after wisdom tooth extraction.

**Table 2:** Odds Ratios for different techniques

	Method A		Method B	OR	CI	*p*-val
Image Noise	DL‑1	Vs	DL-1-SD	0.257	[0.15; 0.44]	< 0.001
	DL‑1	Vs	DL‑2	0.011	[< 0.01; 0.02]	< 0.001
	DL‑1	Vs	AIDR	19.005	[11.56; 31.24]	< 0.001
	DL-1-SD	Vs	DL‑2	0.043	[0.02; 0.08]	< 0.001
	DL-1-SD	Vs	AIDR	74.076	[37.92; 144.70]	< 0.001
	DL‑2	Vs	AIDR	> 999.999	[825.81;> 999.99]	< 0.001
Image sharpness	DL‑1	Vs	DL-1-SD	0.393	[0.25; 0.61]	< 0.001
	DL‑1	Vs	DL‑2	0.072	[0.05; 0.10]	< 0.001
	DL‑1	Vs	AIDR	30.215	[18.85; 48.42]	< 0.001
	DL-1-SD	Vs	DL‑2	0.184	[0.12; 0.28]	< 0.001
	DL-1-SD	Vs	AIDR	76.901	[44.56; 132.70]	< 0.001
	DL‑2	Vs	AIDR	418.204	[252.00; 694.02]	< 0.001
Diagnostic acceptability	DL‑1	Vs	DL-1-SD	0.407	[0.27; 0.61]	< 0.001
	DL‑1	Vs	DL‑2	0.035	[0.02; 0.05]	< 0.001
	DL‑1	Vs	AIDR	25.164	[6.30; 38.85]	< 0.001
	DL-1-SD	Vs	DL‑2	0.086	[0.06; 0.13]	< 0.001
	DL-1-SD	Vs	AIDR	61.760	[37.10; 102.82]	< 0.001
	DL‑2	Vs	AIDR	720.150	[410.14;> 999.99]	< 0.001
Oropharynx	DL‑1	Vs	DL-1-SD	0.601	[0.41; 0.89]	0.011
	DL‑1	Vs	DL‑2	0.085	[0.06; 0.12]	< 0.001
	DL‑1	Vs	AIDR	7.732	[5.28; 11.32]	< 0.001
	DL-1-SD	Vs	DL‑2	0.141	[0.09; 0.22]	< 0.001
	DL-1-SD	Vs	AIDR	12.870	[7.95; 20.83]	< 0.001
	DL‑2	Vs	AIDR	91.200	[55.32; 150.35]	< 0.001
Floor of mouth	DL‑1	Vs	DL-1-SD	0.502	[0.34; 0.74]	< 0.001
	DL‑1	Vs	DL‑2	0.040	[0.03; 0.06]	< 0.001
	DL‑1	Vs	AIDR	15.007	[10.06; 22.40]	< 0.001
	DL-1-SD	Vs	DL‑2	0.079	[0.05; 0.12]	< 0.001
	DL-1-SD	Vs	AIDR	29.915	[18.33; 48.83]	< 0.001
	DL‑2	Vs	AIDR	379.667	[212.65; 677.85]	< 0.001

All statistics based on GEE. Patients and readers were included as random effects. Intercepts omitted for brevity

*OR* Odds ratio; *OR >* *1* Method A is more likely to show a higher rating for given parameter compared to B; *AIDR* Adaptive Iterative Dose Reduction; *DL‑1* Deep-learning Reconstruction; *DL-1-SD* Deep-learning Reconstruction without weight adapted kV-Modulation; *DL‑2* Novel Deep-learning Reconstruction

**Table 3:** Gwents AC for interrater agreement

	Method	κG	CI
Image Noise	AIDR	0.97	[0.958; 0.983]
	DL‑1	0.87	[0.833; 0.901]
	DL-1-SD	0.95	[0.917; 0.98]
	DL‑2	0.97	[0.956; 0.98]
Image sharpness	AIDR	0.97	[0.954; 0.982]
	DL‑1	0.82	[0.763; 0.87]
	DL-1-SD	0.93	[0.905; 0.96]
	DL‑2	0.95	[0.933; 0.961]
Diagnostic acceptability	AIDR	0.97	[0.958; 0.983]
	DL‑1	0.85	[0.815; 0.892]
	DL-1-SD	0.97	[0.95; 0.988]
	DL‑2	0.96	[0.944; 0.971]
Artifacts	AIDR	0.79	[0.738; 0.846]
	DL‑1	0.70	[0.639; 0.767]
	DL-1-SD	0.98	[0.954; 0.999]
	DL‑2	0.57	[0.5; 0.636]
Oropharynx	AIDR	0.97	[0.961; 0.984]
	DL‑1	0.82	[0.774; 0.863]
	DL-1-SD	0.91	[0.854; 0.956]
	DL‑2	0.86	[0.813; 0.899]
Nasopharynx	AIDR	0.98	[0.969; 0.988]
	DL‑1	0.87	[0.828; 0.909]
	DL-1-SD	0.96	[0.933; 0.986]
	DL‑2	0.97	[0.961; 0.982]
Oral cavity	AIDR	0.92	[0.902; 0.946]
	DL‑1	0.76	[0.706; 0.808]
	DL-1-SD	0.77	[0.676; 0.858]
	DL‑2	0.68	[0.601; 0.751]
Floor of mouth	AIDR	0.98	[0.967; 0.99]
	DL‑1	0.84	[0.799; 0.875]
	DL-1-SD	0.96	[0.935; 0.993]
	DL‑2	0.95	[0.928; 0.966]
Salivary glands	AIDR	0.98	[0.967; 0.987]
	DL‑1	0.85	[0.817; 0.891]
	DL-1-SD	0.97	[0.949; 0.988]
	DL‑2	0.95	[0.928; 0.963]
Lymphnodes	AIDR	0.97	[0.96; 0.983]
	DL‑1	0.86	[0.826; 0.9]
	DL-1-SD	0.96	[0.939; 0.983]
	DL‑2	0.95	[0.935; 0.964]

*κ*_*G*_ Gwet’s AC_2_; *CI* Condifence interval; *AIDR* Adaptive Iterative Dose Reduction (AIDR); *DL‑1* Deep-learning Reconstruction with lower dose; *DL-1-SD* Deep-learning Reconstruction with standard dose; *DL‑2* Novel Deep-learning Reconstruction with lower dose

Figures 4 and 5 show examples of an 84 years old female with squamous cell carcinoma of the lower jaw (Fig. 3) and an 18 years old male patient with infection after wisdom tooth extraction (Fig. 4).

The interrater agreement showed excellent values for all defined parameters, especially with regard to image noise (Gwet’s AC_2_ ≥ 0.87), image sharpness (Gwet’s AC_2_ ≥ 0.782) diagnostic acceptability (Gwet’s AC_2_ ≥ 0.85). Detailed values for Gwet’s AC_2_ are listed in Table 3.

**Table 4:** Objective data

Region of interest	Parameter		Estimate	Standard Error	95%-CI	*p*-Value
Floor of mouth	Image noise	AIDR	11.87	0.12	[11.63; 12.11]	–
		DL‑1	−0.37	0.17	[−0.71; −0.04]	0.031
		DL-1-SD	−4.96	0.25	[−5.45; −4.46]	< 0.001
		DL‑2	−6.38	0.17	[−6.72; −6.04]	< 0.001
	SI	AIDR	68.42	1.81	[64.83; 72.01]	–
		DL‑1	2.66	2.57	[−2.40; 7.72]	0.302
		DL-1-SD	−6.10	3.75	[−13.50; 1.29]	0.105
		DL‑2	−0.81	2.57	[−5.87; 4.25]	0.752
	SNR	AIDR	5.92	0.24	[5.43; 6.40]	–
		DL‑1	0.41	0.35	[−0.27; 1.10]	0.234
		DL-1-SD	3.26	0.51	[2.27; 4.26]	< 0.001
		DL‑2	6.63	0.35	[5.94; 7.31]	< 0.001
Masseteric muscle	Image noise	AIDR	14.02	0.16	[13.71; 14.33]	–
		DL‑1	−0.41	0.22	[−0.84; 0.03]	0.067
		DL-1-SD	−5.46	0.32	[−6.09; −4.83]	< 0.001
		DL‑2	−8.04	0.22	[−8.47; −7.61]	< 0.001
	SI	AIDR	77.67	1.58	[74.55; 80.79]	–
		DL‑1	1.95	2.23	[−2.45; 6.35]	0.384
		DL-1-SD	−3.31	3.26	[−9.73; 3.12]	0.312
		DL‑2	−0.63	2.23	[−5.03; 3.77]	0.778
	SNR	AIDR	5.67	0.19	[5.29; 6.05]	–
		DL‑1	0.25	0.27	[−0.28; 0.78]	0.357
		DL-1-SD	3.20	0.40	[2.42; 3.98]	< 0.001
		DL‑2	7.40	0.27	[6.86; 7.93]	< 0.001
	CNR	AIDR	12.15	0.31	[11.52; 12.77]	–
		DL‑1	1.04	0.44	[0.17; 1.92]	0.020
		DL-1-SD	8.37	0.65	[7.09; 9.65]	< 0.001
		DL‑2	15.79	0.44	[14.91; 16.66]	< 0.001
Temporalis	Image noise SI	AIDR	14.47	0.18	[14.11; 14.82]	–
		DL‑1	−0.19	0.25	[−0.69; 0.30]	0.445
		DL-1-SD	−5.40	0.37	[−6.13; −4.68]	< 0.001
		DL‑2	−7.83	0.25	[−8.32; −7.33]	< 0.001
		AIDR	65.88	1.47	[62.96; 68.79]	–
		DL‑1	2.49	2.08	[−1.61; 6.60]	0.233
		DL-1-SD	−0.52	3.04	[−6.52; 5.48]	0.865
		DL‑2	3.59	2.08	[−0.52; 7.70]	0.086
	CNR	AIDR	10.77	0.29	[10.19; 11.34]	–
		DL‑1	0.68	0.41	[−0.13; 1.49]	0.099
		DL-1-SD	6.22	0.60	[5.03; 7.40]	< 0.001
		DL‑2	13.38	0.41	[12.56; 14.19]	< 0.001
	SNR	AIDR	4.70	0.19	[4.32; 5.09]	–
		DL‑1	0.18	0.28	[−0.36; 0.72]	0.513
		DL-1-SD	2.78	0.40	[1.98; 3.57]	< 0.001
		DL‑2	6.10	0.28	[5.56; 6.64]	< 0.001

All statistics based on Mixed Models for Repeated Measures (MMRM). Patients were included as random effects. Based on score test with the null hypothesis of effect estimates being zero

*AIDR* Adaptive Iterative Dose Reduction; *DL‑1* Deep-learning Reconstruction with lower dose; *DL-1-SD* Deep-learning Reconstruction with standard dose; *DL‑2* Novel Deep-learning Reconstruction with lower dose

### Objective Image Quality

The assessment of objective image criteria demonstrated that similar to subjective image quality DL‑2 significantly outmatched plain AIDR and DL‑1 algorithm for all defined parameters and regions (image noise, masseteric muscle with 14.02 [AIDR] vs. 13.61 [DL-1]: *p* = 0.067 and with 5.98 [DL-2]: *p* < 0.001; CNR with 12.15 [AIDR] vs. 13.19 [DL-1]: *p* = 0.020 and with 27.94 [DL-2]: *p* < 0.001), showing similar results for all three anatomic location (Table 4). Thereby, AiCE did not affect signal intensity values.

### Radiation Dose

Values of CTDIvol, DLP, and mean effective dose in msv were compared to evaluate dose exposure. Using weight-adapted kV reduction with tube current modulation while simultaneously applying AiCE, very low radiation doses could be achieved (CTDIvol: 7.4 ± 4.2 mGy). Calculating DLP with an approximated mean scan length of 25 cm resulted in a mean DLP of 185 ± 105 mGy*cm and a mean effective dose of 1.07 (± 0.58) mSv. Radiation dose was significantly lower than without weight-adapted kV reduction (CTDIvol: 10.16 ± 2.0 mGy; DLP: 254 ± 50 mGy*cm; *p* < 0.001).

## Discussion

This study is the first to evaluate the tube current modulation and body weight-adapted tube voltage in combination with a novel, recently implemented deep-learning-based reconstruction algorithm. Thereby we aimed to investigate the advantages of UHR-CT combined with a new deep learning-based image reconstruction engine, for head and neck imaging. Our analysis revealed that DL‑2 reconstruction significantly improves image quality and diagnostic confidence, by markedly increasing SNR and CNR while simultaneously significantly decreasing radiation exposure through kV-modulation integration. Thereby, we could achieve very low radiation doses with a mean CTDIvol of 7.4 mGy, undercutting radiation exposure compared to the former standard protocol by 27.2% and current diagnostic reference values by about 50%, still demonstrating excellent image quality [13, 16, 20, 21].

Iterative Reconstruction algorithms have become a state-of-the-art technique, significantly reducing radiation dose compared to filtered back projection techniques [22, 23]. Currently, with deep learning-based image reconstruction, a new era of post-processing is evolving, resulting in even better image quality, while it seems that further reduction of radiation exposure is possible [12, 24]. By combining the potential benefits of tube current modulation and reduced kV levels with deep learning-based image reconstruction through AiCE, we could not only generate high-quality scans but furthermore reduced kV results in greater iodine signal which may improve tumor depiction [25–27]. Additionally, regarding radiation protection and following the principle of “as low as reasonable achievable” (ALARA) innovative instruments for reduction of dose-volume need to be identified. Therefore, our described algorithm will be implemented into everyday clinical practice.

In evaluating a new neck mass in adults, using CT as the primary imaging modality is standard practice. This is due to its capacity for fast and comprehensive assessment of all neck tissues, widespread availability, and lower cost compared to MRI [3]. Furthermore, in the emergency department, CT scans are often used in the initial assessment to check for infectious diseases in the head and neck area [28]. Generating high-quality CT images and enabling accurate diagnosis is crucial. However, with the growing number of radiation exposure, especially due to repetitive CT scans related to longer life expectancies and improved cancer survival rates, it is important to decrease applied radiation doses to a reasonable minimum [29–31]. This technique is highly relevant and well-suited for young patients requiring emergency imaging. Moreover, patients undergoing repeated CT examinations due to contraindications for MRI may benefit from reduced radiation exposure. Also, hypervascularized structures may be visualized more effectively due to the lower kV settings.

This study demonstrates, that by combining advantages in hardware with advanced deep learning reconstruction algorithms, it is possible not only to reduce radiation exposure but to simultaneously improve image quality, while generating excellent image quality.

However this study has some limitations. As it represents a single-centered, retrospective study, it is associated with selection bias. Furthermore, we did not evaluate the diagnostic accuracy of particular diseases. The primary focus was on image quality. Reconstruction techniques were chosen to compare the former standard technique of AIDR with currently available AI-based reconstruction techniques for Aquillion precision. We did not focus on other reconstruction techniques offered by other vendors. Therefore, future prospective studies are essential to address these limitations and to evaluate the impact of radiation dose reduction combined with AI-based reconstruction algorithms for specific diseases and different vendors.

## Conclusion

Employing AI-based reconstruction algorithms in ultra-high resolution head and neck imaging ensures high image quality while achieving very low radiation exposure, thereby enhancing the scope of diagnostic imaging.

## Supplementary Information

Online-Appendix

## Abbreviations

AiCE: Advanced intelligent Clear-IQ Engine
AIDR: Adaptive iterative dose reduction
CNR: Contrast-to-Noise Ratio
DL‑1: Established AiCE-Body reconstruction with dose reduction
DL‑2: Recently developed reconstruction algorithm AiCE-CTA with dose reduction
DL-1-SD: Established AiCE-Body reconstruction with standard dose
MBIR: Model-based iterative reconstruction
ROI: Region of interest
SNR: Signal-to-Noise Ratio
