# Supplementary material for: Radiation Dose Reduction and Image Quality Improvement of UHR CT of the Neck by Novel Deep-learning Image Reconstruction
Source: Clin Neuroradiol. 2025 Jun 30;35(4):755–65. doi: 10.1007/s00062-025-01532-5 (PMC12552282; doi:10.1007/s00062-025-01532-5)
Supplement: Supplementary file 1 — Online-Appendix [file 62_2025_1532_MOESM1_ESM.docx]

**Supplementary Tables**

| Table S1 Patient characteristics |  |
| --- | --- |
| *Characteristics* | **Value (n=30)** |
| *Age (years)** | 67.5 ± 10.4 (47-88) |
| *Median age* | 67.5 |
| *Sex* |  |
| *Male* | 15 (50 %) |
| *Female* | 15 (50%) |
| *Weight (kg)* | 75.6 ± 16 (52-130) |
| *Pathologic findings* |  |
| *Squamous cell carcinoma* | 27 (90%) |
| *lower jaw* | 10 (33.3%) |
| *tongue* | 9 (30.0%) |
| *floor of the mouth* | 5 (16.6%) |
| *upper jaw* | 3 (10.0%) |
| *palate* | 2 (6.6%) |
| *Basal-cell carcinoma* | 1 (3.3%) |
| ** Data are mean ± 1SD, with ranges in parentheses* |  |
|  |  |

**Table S2 Likert-scale for subjective image evaluation**

| Grading | Image noise | Image sharpness | Artefacts | Diagnostic acceptability |
| --- | --- | --- | --- | --- |
| 1 | Unacceptable | Blurry | Present and affecting image interpretation | Unacceptable |
| 2 | Increased | Poorer than average | Present and affecting visualization of normal structures | Suboptimal |
| 3 | Average | Subtle lesion | Present but not affecting visualization of normal structures | Average |
| 4 | Less than average | Clearly visualized lesion, poor margin | None | Above average |
| 5 | Minimum or no noise | Clearly visualized lesion, clearly visualized margin |  | superior |

| \| **Table S3 Reported overall subjective image quality** \| \| \| \| \| \| \| \| \| \| \| \| \| \| \| \| \| \| \| \| \| \| \| \| \| \| \| \| \| \| \| \| \| \| \| \| \| \| \| \| \| \| \| \| \| \| \| \| \| \| \| \| \| \| \| \| \| --- \| --- \| --- \| --- \| --- \| --- \| --- \| --- \| --- \| --- \| --- \| --- \| --- \| --- \| --- \| --- \| --- \| --- \| --- \| --- \| --- \| --- \| --- \| --- \| --- \| --- \| --- \| --- \| --- \| --- \| --- \| --- \| --- \| --- \| --- \| --- \| --- \| --- \| --- \| --- \| --- \| --- \| --- \| --- \| --- \| --- \| --- \| --- \| --- \| --- \| --- \| --- \| --- \| --- \| --- \| --- \| \|  \| \|  \| \| \| \| \| \| \| **Reader 1** \| \| \| \| \| \| \| \| \| \| \| \| \| \| \| \| \| \| \| \| \| \| \| \| **Reader 2** \| \| \| \| \| \| \| \| \| \| \| \| \| \| \| \| \| \| \| \| \| \| \| \| \| **Parameter** \| \| \| \| \| \| \| **AIDR** \| \| \| \| \| \| **DL-1** \| \| \| \| \| \| **DL-1-SD** \| \| \| \| \| \| **DL-2** \| \| \| \| \| \| **AIDR** \| \| \| \| \| \| **DL-1** \| \| \| \| \| \| **DL-1-SD** \| \| \| \| \| \| **DL-2** \| \| \| \| \| \| \|  \| \| \| \| \| \| \| **n** \| \| \| **(%)** \| \| \| **n** \| \| \| **(%)** \| \| \| **n** \| \| \| **(%)** \| \| \| **n** \| \| \| **(%)** \| \| \| **n** \| \| \| **(%)** \| \| \| **n** \| \| \| **(%)** \| \| \| **n** \| \| \| **(%)** \| \| \| **n** \| \| \| **(%)** \| \| \| \| Image Noise \| \| \| \| 5 \| \| \| 2 \| \| \| (2.0) \| \| \| 0 \| \| \| (0.0) \| \| \| 5 \| \| \| (16.7) \| \| \| 81 \| \| \| (82.7) \| \| \| 0 \| \| \| (0.0) \| \| \| 9 \| \| \| (9.2) \| \| \| 1 \| \| \| (3.3) \| \| \| 74 \| \| \| (75.5) \| \| \| \| 4 \| \| \| 8 \| \| \| (8.2) \| \| \| 41 \| \| \| (41.8) \| \| \| 19 \| \| \| (63.3) \| \| \| 17 \| \| \| (17.3) \| \| \| 7 \| \| \| (7.1) \| \| \| 74 \| \| \| (75.5) \| \| \| 28 \| \| \| (93.3) \| \| \| 24 \| \| \| (24.5) \| \| \| \| 3 \| \| \| 85 \| \| \| (86.7) \| \| \| 47 \| \| \| (48.0) \| \| \| 6 \| \| \| (20.0) \| \| \| 0 \| \| \| (0.0) \| \| \| 89 \| \| \| (90.8) \| \| \| 14 \| \| \| (14.3) \| \| \| 1 \| \| \| (3.3) \| \| \| 0 \| \| \| (0.0) \| \| \| \| 2 \| \| \| 3 \| \| \| (3.1) \| \| \| 10 \| \| \| (10.2) \| \| \| 0 \| \| \| (0.0) \| \| \| 0 \| \| \| (0.0) \| \| \| 2 \| \| \| (2.0) \| \| \| 1 \| \| \| (1.0) \| \| \| 0 \| \| \| (0.0) \| \| \| 0 \| \| \| (0.0) \| \| \| \| 1 \| \| \| 0 \| \| \| (0.0) \| \| \| 0 \| \| \| (0.0) \| \| \| 0 \| \| \| (0.0) \| \| \| 0 \| \| \| (0.0) \| \| \| 0 \| \| \| (0.0) \| \| \| 0 \| \| \| (0.0) \| \| \| 0 \| \| \| (0.0) \| \| \| 0 \| \| \| (0.0) \| \| \| \|  \| \| \| \| \| \| \| \| \| \| \| \| \| \| \| \| \| \| \| \| \| \| \| \| \| \| \| \| \| \| \| \| \| \| \| \| \| \| \| \| \| \| \| \| \| \| \| \| \| \| \| \| \| \| \| \| Image sharpness \| \| \| \| 5 \| \| \| 1 \| \| \| (1.0) \| \| \| 0 \| \| \| (0.0) \| \| \| 8 \| \| \| (26.7) \| \| \| 75 \| \| \| (76.5) \| \| \| 1 \| \| \| (1.0) \| \| \| 14 \| \| \| (14.3) \| \| \| 0 \| \| \| (0.0) \| \| \| 53 \| \| \| (54.1) \| \| \| \| 4 \| \| \| 12 \| \| \| (2.2) \| \| \| 47 \| \| \| (48.0) \| \| \| 21 \| \| \| (70.0) \| \| \| 23 \| \| \| (23.5) \| \| \| 7 \| \| \| (7.1) \| \| \| 78 \| \| \| (79.6) \| \| \| 29 \| \| \| (96.7) \| \| \| 45 \| \| \| (45.9) \| \| \| \| 3 \| \| \| 83 \| \| \| (84.7) \| \| \| 47 \| \| \| (48.0) \| \| \| 1 \| \| \| (3.3) \| \| \| 0 \| \| \| (0.0) \| \| \| 89 \| \| \| (90.8) \| \| \| 6 \| \| \| (6.1) \| \| \| 1 \| \| \| (3.3) \| \| \| 0 \| \| \| (0.0) \| \| \| \| 2 \| \| \| 2 \| \| \| (2.0) \| \| \| 4 \| \| \| (4.1) \| \| \| 0 \| \| \| (0.0) \| \| \| 0 \| \| \| (0.0) \| \| \| 1 \| \| \| (1.0) \| \| \| 0 \| \| \| (0.0) \| \| \| 0 \| \| \| (0.0) \| \| \| 0 \| \| \| (0.0) \| \| \| \| 1 \| \| \| 0 \| \| \| (0.0) \| \| \| 0 \| \| \| (0.0) \| \| \| 0 \| \| \| (0.0) \| \| \| 0 \| \| \| (0.0) \| \| \| 0 \| \| \| (0.0) \| \| \| 0 \| \| \| (0.0) \| \| \| 0 \| \| \| (0.0) \| \| \| 0 \| \| \| (0.0) \| \| \| \|  \| \| \| \| \| \| \| \| \| \| \| \| \| \| \| \| \| \| \| \| \| \| \| \| \| \| \| \| \| \| \| \| \| \| \| \| \| \| \| \| \| \| \| \| \| \| \| \| \| \| \| \| \| \| \| \| Diagnostic acceptability \| \| \| \| 5 \| \| \| 0 \| \| \| (0.0) \| \| \| 0 \| \| \| (0.0) \| \| \| 6 \| \| \| (20.0) \| \| \| 71 \| \| \| (72.4) \| \| \| 0 \| \| \| (0.0) \| \| \| 9 \| \| \| (9.2) \| \| \| 0 \| \| \| (0.0) \| \| \| 67 \| \| \| (68.4) \| \| \| \| 4 \| \| \| 12 \| \| \| (12.2) \| \| \| 54 \| \| \| (55.1) \| \| \| 24 \| \| \| (80.0) \| \| \| 27 \| \| \| (27.6) \| \| \| 10 \| \| \| (10.2) \| \| \| 74 \| \| \| (75.5) \| \| \| 30 \| \| \| (100.0) \| \| \| 30 \| \| \| (30.6) \| \| \| \|  \| \| \| \| 3 \| \| \| 86 \| \| \| (87.8) \| \| \| 41 \| \| \| (41.8) \| \| \| 0 \| \| \| (0.0) \| \| \| 0 \| \| \| (0.0) \| \| \| 87 \| \| \| (88.8) \| \| \| 15 \| \| \| (15.3) \| \| \| 0 \| \| \| (0.0) \| \| \| 1 \| \| \| (1.0) \| \| \| \|  \| \| \| \| 2 \| \| \| 0 \| \| \| (0.0) \| \| \| 3 \| \| \| (3.1) \| \| \| 0 \| \| \| (0.0) \| \| \| 0 \| \| \| (0.0) \| \| \| 1 \| \| \| (1.0) \| \| \| 0 \| \| \| (0.0) \| \| \| 0 \| \| \| (0.0) \| \| \| 0 \| \| \| (0.0) \| \| \| \|  \| \| \| \| 1 \| \| \| 0 \| \| \| (0.0) \| \| \| 0 \| \| \| (0.0) \| \| \| 0 \| \| \| (0.0) \| \| \| 0 \| \| \| (0.0) \| \| \| 0 \| \| \| (0.0) \| \| \| 0 \| \| \| (0.0) \| \| \| 0 \| \| \| (0.0) \| \| \| 0 \| \| \| (0.0) \| \| \| \|  \| \| \| \|  \| \| \|  \| \| \|  \| \| \|  \| \| \|  \| \| \|  \| \| \|  \| \| \|  \| \| \|  \| \| \|  \| \| \|  \| \| \|  \| \| \|  \| \| \|  \| \| \|  \| \| \|  \| \| \|  \| \| \| \| Artifacts \| \| \| \| 5 \| \| \| 6 \| \| \| (6.1) \| \| \| 0 \| \| \| (0.0) \| \| \| 0 \| \| \| (0.0) \| \| \| 6 \| \| \| (6.1) \| \| \| 1 \| \| \| (1.0) \| \| \| 7 \| \| \| (7.1) \| \| \| 0 \| \| \| (0.0) \| \| \| 21 \| \| \| (21.4) \| \| \| \|  \| \| \| \| 4 \| \| \| 23 \| \| \| (23.5) \| \| \| 23 \| \| \| (23.5) \| \| \| 7 \| \| \| (23.3) \| \| \| 15 \| \| \| (15.3) \| \| \| 15 \| \| \| (15.3) \| \| \| 15 \| \| \| (15.3) \| \| \| 7 \| \| \| (23.3) \| \| \| 19 \| \| \| (19.4) \| \| \| \|  \| \| \| \| 3 \| \| \| 28 \| \| \| (28.6) \| \| \| 29 \| \| \| (29.6) \| \| \| 6 \| \| \| (20.0) \| \| \| 31 \| \| \| (31.6) \| \| \| 77 \| \| \| (78.6) \| \| \| 51 \| \| \| (52.0) \| \| \| 6 \| \| \| (20.0) \| \| \| 23 \| \| \| (23.5) \| \| \| \|  \| \| \| \| 2 \| \| \| 41 \| \| \| (41.8) \| \| \| 46 \| \| \| (46.9) \| \| \| 17 \| \| \| (56.7) \| \| \| 46 \| \| \| (46.9) \| \| \| 5 \| \| \| (5.1) \| \| \| 25 \| \| \| (25.5) \| \| \| 17 \| \| \| (56.7) \| \| \| 35 \| \| \| (35.7) \| \| \| \|  \| \| \| \| 1 \| \| \| 0 \| \| \| (0.0) \| \| \| 0 \| \| \| (0.0) \| \| \| 0 \| \| \| (0.0) \| \| \| 0 \| \| \| (0.0) \| \| \| 0 \| \| \| (0.0) \| \| \| 0 \| \| \| (0.0) \| \| \| 0 \| \| \| (0.0) \| \| \| 0 \| \| \| (0.0) \| \| \| \|  \| \| \| \|  \| \| \|  \| \| \|  \| \| \|  \| \| \|  \| \| \|  \| \| \|  \| \| \|  \| \| \|  \| \| \|  \| \| \|  \| \| \|  \| \| \|  \| \| \|  \| \| \|  \| \| \|  \| \| \|  \| \| \| \| Oropharynx \| \| \| \| 5 \| \| \| 0 \| \| \| (0.0) \| \| \| 0 \| \| \| (0.0) \| \| \| 2 \| \| \| (6.7) \| \| \| 62 \| \| \| (63.3) \| \| \| 0 \| \| \| (0.0) \| \| \| 6 \| \| \| (6.1) \| \| \| 0 \| \| \| (0.0) \| \| \| 46 \| \| \| (46.9) \| \| \| \|  \| \| \| \| 4 \| \| \| 7 \| \| \| (7.1) \| \| \| 27 \| \| \| (27.6) \| \| \| 16 \| \| \| (53.3) \| \| \| 21 \| \| \| (21.4) \| \| \| 8 \| \| \| (8.2) \| \| \| 53 \| \| \| (54.1) \| \| \| 17 \| \| \| (56.7) \| \| \| 31 \| \| \| (31.6) \| \| \| \|  \| \| \| \| 3 \| \| \| 80 \| \| \| (81.6) \| \| \| 66 \| \| \| (67.3) \| \| \| 11 \| \| \| (36.7) \| \| \| 13 \| \| \| (13.3) \| \| \| 81 \| \| \| (82.7) \| \| \| 36 \| \| \| (36.7) \| \| \| 12 \| \| \| (40.0) \| \| \| 17 \| \| \| (17.3) \| \| \| \|  \| \| \| \| 2 \| \| \| 11 \| \| \| (11.2) \| \| \| 5 \| \| \| (5.1) \| \| \| 1 \| \| \| (3.3) \| \| \| 2 \| \| \| (2.0) \| \| \| 9 \| \| \| (9.2) \| \| \| 3 \| \| \| (3.1) \| \| \| 1 \| \| \| (3.3) \| \| \| 4 \| \| \| (4.1) \| \| \| \|  \| \| \| \| 1 \| \| \| 0 \| \| \| (0.0) \| \| \| 0 \| \| \| (0.0) \| \| \| 0 \| \| \| (0.0) \| \| \| 0 \| \| \| (0.0) \| \| \| 0 \| \| \| (0.0) \| \| \| 0 \| \| \| (0.0) \| \| \| 0 \| \| \| (0.0) \| \| \| 0 \| \| \| (0.0) \| \| \| \|  \| \| \| \|  \| \| \|  \| \| \|  \| \| \|  \| \| \|  \| \| \|  \| \| \|  \| \| \|  \| \| \|  \| \| \|  \| \| \|  \| \| \|  \| \| \|  \| \| \|  \| \| \|  \| \| \|  \| \| \|  \| \| \| \| Nasopharynx \| \| \| \| 5 \| \| \| 0 \| \| \| (0.0) \| \| \| 0 \| \| \| (0.0) \| \| \| 8 \| \| \| (26.7) \| \| \| 81 \| \| \| (82.7) \| \| \| 0 \| \| \| (0.0) \| \| \| 9 \| \| \| (9.2) \| \| \| 0 \| \| \| (0.0) \| \| \| 76 \| \| \| (77.6) \| \| \| \|  \| \| \| \| 4 \| \| \| 11 \| \| \| (11.2) \| \| \| 47 \| \| \| (48.0) \| \| \| 20 \| \| \| (66.7) \| \| \| 16 \| \| \| (16.3) \| \| \| 12 \| \| \| (12.2) \| \| \| 83 \| \| \| (84.7) \| \| \| 30 \| \| \| (100.0) \| \| \| 22 \| \| \| (22.4) \| \| \| \|  \| \| \| \| 3 \| \| \| 87 \| \| \| (88.8) \| \| \| 49 \| \| \| (50.0) \| \| \| 2 \| \| \| (6.7) \| \| \| 1 \| \| \| (1.0) \| \| \| 85 \| \| \| (86.7) \| \| \| 6 \| \| \| (6.1) \| \| \| 0 \| \| \| (0.0) \| \| \| 0 \| \| \| (0.0) \| \| \| \|  \| \| \| \| 2 \| \| \| 0 \| \| \| (0.0) \| \| \| 2 \| \| \| (2.0) \| \| \| 0 \| \| \| (0.0) \| \| \| 0 \| \| \| (0.0) \| \| \| 1 \| \| \| (1.0) \| \| \| 0 \| \| \| ( 0.0) \| \| \| 0 \| \| \| (0.0) \| \| \| 0 \| \| \| (0.0) \| \| \| \|  \| \| \| \| 1 \| \| \| 0 \| \| \| (0.0) \| \| \| 0 \| \| \| (0.0) \| \| \| 0 \| \| \| (0.0) \| \| \| 0 \| \| \| (0.0) \| \| \| 0 \| \| \| (0.0) \| \| \| 0 \| \| \| ( 0.0) \| \| \| 0 \| \| \| (0.0) \| \| \| 0 \| \| \| (0.0) \| \| \| \|  \| \| \| \|  \| \| \|  \| \| \|  \| \| \|  \| \| \|  \| \| \|  \| \| \|  \| \| \|  \| \| \|  \| \| \|  \| \| \|  \| \| \|  \| \| \|  \| \| \|  \| \| \|  \| \| \|  \| \| \|  \| \| \| \|  \| \| \| \| \| \| \| **Reader 3** \| \| \| \| \| \| \| \| \| \| \| \| \| \| \| \| \| \| \| \| \| \| \| \| **Overall** \| \| \| \| \| \| \| \| \| \| \| \| \| \| \| \| \| \| \| \| \| \| \| \| \| **Parameter** \| \| \| \| \| \| \| **AIDR** \| \| \| \| \| \| **DL-1** \| \| \| \| \| \| **DL-1-SD** \| \| \| \| \| \| **DL-2** \| \| \| \| \| \| **AIDR** \| \| \| \| \| \| **DL-1** \| \| \| \| \| \| **DL-1-SD** \| \| \| \| \| \| **DL-2** \| \| \| \| \| \| \|  \| \| \| \| \| \| \| **n** \| \| \| **(%)** \| \| \| **n** \| \| \| **(%)** \| \| \| **n** \| \| \| **(%)** \| \| \| **n** \| \| \| **(%)** \| \| \| **n** \| \| \| **(%)** \| \| \| **n** \| \| \| **(%)** \| \| \| **n** \| \| \| **(%)** \| \| \| **n** \| \| \| **(%)** \| \| \| \| Image Noise \| \| \| \| 5 \| \| \| 0 \| \| \| (0.0) \| \| \| 3 \| \| \| (3.1) \| \| \| 8 \| \| \| (26.7) \| \| \| 86 \| \| \| (87.8) \| \| \| 2 \| \| \| (0.7) \| \| \| 12 \| \| \| (4.1) \| \| \| 14 \| \| \| (15.6) \| \| \| 241 \| \| \| (82.0) \| \| \| \|  \| \| \| \| 4 \| \| \| 4 \| \| \| (4.1) \| \| \| 78 \| \| \| (79.6) \| \| \| 20 \| \| \| (66.7) \| \| \| 9 \| \| \| (9.2) \| \| \| 19 \| \| \| (6.5) \| \| \| 193 \| \| \| (65.6) \| \| \| 67 \| \| \| (74.4) \| \| \| 50 \| \| \| (17.0) \| \| \| \|  \| \| \| \| 3 \| \| \| 84 \| \| \| (85.7) \| \| \| 17 \| \| \| (17.3) \| \| \| 2 \| \| \| (6.7) \| \| \| 3 \| \| \| (3.1) \| \| \| 258 \| \| \| (87.8) \| \| \| 78 \| \| \| (26.5) \| \| \| 9 \| \| \| (10.0) \| \| \| 3 \| \| \| (1.0) \| \| \| \|  \| \| \| \| 2 \| \| \| 10 \| \| \| (10.2) \| \| \| 0 \| \| \| (0.0) \| \| \| 0 \| \| \| (0.0) \| \| \| 0 \| \| \| (0.0) \| \| \| 15 \| \| \| (5.1) \| \| \| 11 \| \| \| (3.7) \| \| \| 0 \| \| \| (0.0) \| \| \| 0 \| \| \| (0.0) \| \| \| \|  \| \| \| \| 1 \| \| \| 0 \| \| \| (0.0) \| \| \| 0 \| \| \| (0.0) \| \| \| 0 \| \| \| (0.0) \| \| \| 0 \| \| \| (0.0) \| \| \| 0 \| \| \| (0.0) \| \| \| 0 \| \| \| (0.0) \| \| \| 0 \| \| \| (0.0) \| \| \| 0 \| \| \| (0.0) \| \| \| \|  \| \| \| \|  \| \| \|  \| \| \|  \| \| \|  \| \| \|  \| \| \|  \| \| \|  \| \| \|  \| \| \|  \| \| \|  \| \| \|  \| \| \|  \| \| \|  \| \| \|  \| \| \|  \| \| \|  \| \| \|  \| \| \| \| Image sharpness \| \| \| \| 5 \| \| \| 0 \| \| \| (0.0) \| \| \| 38 \| \| \| (38.8) \| \| \| 15 \| \| \| (50.0) \| \| \| 78 \| \| \| (79.6) \| \| \| 2 \| \| \| (0.7) \| \| \| 52 \| \| \| (17.7) \| \| \| 23 \| \| \| (25.6) \| \| \| 206 \| \| \| (70.1) \| \| \| \| 4 \| \| \| 11 \| \| \| (11.2) \| \| \| 52 \| \| \| (53.1) \| \| \| 15 \| \| \| (50.0) \| \| \| 19 \| \| \| (19.4) \| \| \| 30 \| \| \| (10.2) \| \| \| 177 \| \| \| (60.2) \| \| \| 65 \| \| \| (72.2) \| \| \| 87 \| \| \| (29.6) \| \| \| \| 3 \| \| \| 82 \| \| \| (83.7) \| \| \| 8 \| \| \| (8.2) \| \| \| 0 \| \| \| (0.0) \| \| \| 1 \| \| \| (1.0) \| \| \| 254 \| \| \| (86.4) \| \| \| 61 \| \| \| (20.7) \| \| \| 2 \| \| \| (2.2) \| \| \| 1 \| \| \| (0.3) \| \| \| \| 2 \| \| \| 5 \| \| \| (5.1) \| \| \| 0 \| \| \| (0.0) \| \| \| 0 \| \| \| (0.0) \| \| \| 0 \| \| \| (0.0) \| \| \| 8 \| \| \| (2.7) \| \| \| 4 \| \| \| (1.4) \| \| \| 0 \| \| \| (0.0) \| \| \| 0 \| \| \| (0.0) \| \| \| \| 1 \| \| \| 0 \| \| \| (0.0) \| \| \| 0 \| \| \| (0.0) \| \| \| 0 \| \| \| (0.0) \| \| \| 0 \| \| \| (0.0) \| \| \| 0 \| \| \| (0.0) \| \| \| 0 \| \| \| (0.0) \| \| \| 0 \| \| \| (0.0) \| \| \| 0 \| \| \| (0.0) \| \| \| \|  \| \| \| \| \| \| \| \| \| \| \| \| \| \| \| \| \| \| \| \| \| \| \| \| \| \| \| \| \| \| \| \| \| \| \| \| \| \| \| \| \| \| \| \| \| \| \| \| \| \| \| \| \| \| \| \| Diagnostic acceptability \| \| \| \| 5 \| \| \| 1 \| \| \| (1.0) \| \| \| 30 \| \| \| (30.6) \| \| \| 6 \| \| \| (20.0) \| \| \| 86 \| \| \| (87.8) \| \| \| 1 \| \| \| (0.3) \| \| \| 39 \| \| \| (13.3) \| \| \| 12 \| \| \| (13.3) \| \| \| 224 \| \| \| (76.2) \| \| \| \| 4 \| \| \| 14 \| \| \| (14.3) \| \| \| 59 \| \| \| (60.2) \| \| \| 24 \| \| \| (80.0) \| \| \| 11 \| \| \| (11.2) \| \| \| 36 \| \| \| (12.2) \| \| \| 187 \| \| \| (63.6) \| \| \| 78 \| \| \| (86.7) \| \| \| 68 \| \| \| (23.1) \| \| \| \| 3 \| \| \| 78 \| \| \| ( 79.6) \| \| \| 9 \| \| \| (9.2) \| \| \| 0 \| \| \| (0.0) \| \| \| 1 \| \| \| (1.0) \| \| \| 251 \| \| \| (85.4) \| \| \| 65 \| \| \| (22.1) \| \| \| 0 \| \| \| (0.0) \| \| \| 2 \| \| \| (0.7) \| \| \| \| 2 \| \| \| 5 \| \| \| (5.1) \| \| \| 0 \| \| \| (0.0) \| \| \| 0 \| \| \| (0.0) \| \| \| 0 \| \| \| (0.0) \| \| \| 6 \| \| \| (2.0) \| \| \| 3 \| \| \| (1.0) \| \| \| 0 \| \| \| (0.0) \| \| \| 0 \| \| \| (0.0) \| \| \| \| 1 \| \| \| 0 \| \| \| (0.0) \| \| \| 0 \| \| \| (0.0) \| \| \| 0 \| \| \| (0.0) \| \| \| 0 \| \| \| (0.0) \| \| \| 0 \| \| \| (0.0) \| \| \| 0 \| \| \| (0.0) \| \| \| 0 \| \| \| (0.0) \| \| \| 0 \| \| \| (0.0) \| \| \| \|  \| \| \| \|  \| \| \|  \| \| \|  \| \| \|  \| \| \|  \| \| \|  \| \| \|  \| \| \|  \| \| \|  \| \| \|  \| \| \|  \| \| \|  \| \| \|  \| \| \|  \| \| \|  \| \| \|  \| \| \|  \| \| \| \| Artifacts \| \| \| \| 5 \| \| \| 17 \| \| \| (17.3) \| \| \| 19 \| \| \| (19.4) \| \| \| 0 \| \| \| (0.0) \| \| \| 44 \| \| \| (44.9) \| \| \| 24 \| \| \| (8.2) \| \| \| 26 \| \| \| (8.8) \| \| \| 0 \| \| \| (0.0) \| \| \| 71 \| \| \| (24.1) \| \| \| \|  \| \| \| \| 4 \| \| \| 21 \| \| \| (21.4) \| \| \| 25 \| \| \| (25.5) \| \| \| 6 \| \| \| (20.0) \| \| \| 20 \| \| \| (20.4) \| \| \| 59 \| \| \| (20.1) \| \| \| 63 \| \| \| (21.4) \| \| \| 20 \| \| \| (22.2) \| \| \| 54 \| \| \| (18.4) \| \| \| \|  \| \| \| \| 3 \| \| \| 50 \| \| \| (51.0) \| \| \| 40 \| \| \| (40.8) \| \| \| 7 \| \| \| (23.3) \| \| \| 30 \| \| \| (30.6) \| \| \| 155 \| \| \| (52.7) \| \| \| 120 \| \| \| (40.8) \| \| \| 19 \| \| \| (21.1) \| \| \| 84 \| \| \| (28.6) \| \| \| \|  \| \| \| \| 2 \| \| \| 10 \| \| \| (10.2) \| \| \| 14 \| \| \| (14.3) \| \| \| 17 \| \| \| (56.7) \| \| \| 4 \| \| \| (4.1) \| \| \| 56 \| \| \| (19.0) \| \| \| 85 \| \| \| (28.9) \| \| \| 51 \| \| \| (56.7) \| \| \| 85 \| \| \| (28.9) \| \| \| \|  \| \| \| \| 1 \| \| \| 0 \| \| \| (0.0) \| \| \| 0 \| \| \| (0.0) \| \| \| 0 \| \| \| (0.0) \| \| \| 0 \| \| \| (0.0) \| \| \| 0 \| \| \| (0.0) \| \| \| 0 \| \| \| (0.0) \| \| \| 0 \| \| \| (0.0) \| \| \| 0 \| \| \| (0.0) \| \| \| \|  \| \| \| \|  \| \| \|  \| \| \|  \| \| \|  \| \| \|  \| \| \|  \| \| \|  \| \| \|  \| \| \|  \| \| \|  \| \| \|  \| \| \|  \| \| \|  \| \| \|  \| \| \|  \| \| \|  \| \| \|  \| \| \| \| Oropharynx \| \| \| \| 5 \| \| \| 1 \| \| \| (1.0) \| \| \| 22 \| \| \| (22.4) \| \| \| 5 \| \| \| (16.7) \| \| \| 76 \| \| \| (77.6) \| \| \| 1 \| \| \| (0.3) \| \| \| 28 \| \| \| (9.5) \| \| \| 7 \| \| \| (7.8) \| \| \| 184 \| \| \| (62.6) \| \| \| \| 4 \| \| \| 10 \| \| \| (10.2) \| \| \| 50 \| \| \| (51.0) \| \| \| 25 \| \| \| (83.3) \| \| \| 19 \| \| \| (19.4) \| \| \| 25 \| \| \| (8.5) \| \| \| 130 \| \| \| (44.2) \| \| \| 58 \| \| \| (64.4) \| \| \| 71 \| \| \| (24.1) \| \| \| \| 3 \| \| \| 82 \| \| \| (83.7) \| \| \| 24 \| \| \| (24.5) \| \| \| 0 \| \| \| (0.0) \| \| \| 3 \| \| \| (3.1) \| \| \| 243 \| \| \| (82.7) \| \| \| 126 \| \| \| (42.9) \| \| \| 23 \| \| \| (25.6) \| \| \| 33 \| \| \| (11.2) \| \| \| \| 2 \| \| \| 5 \| \| \| (5.1) \| \| \| 2 \| \| \| (2.0) \| \| \| 0 \| \| \| (0.0) \| \| \| 0 \| \| \| (0.0) \| \| \| 25 \| \| \| (8.5) \| \| \| 10 \| \| \| (3.4) \| \| \| 2 \| \| \| (2.2) \| \| \| 6 \| \| \| (2.0) \| \| \| \| 1 \| \| \| 0 \| \| \| (0.0) \| \| \| 0 \| \| \| (0.0) \| \| \| 0 \| \| \| (0.0) \| \| \| 0 \| \| \| (0.0) \| \| \| 0 \| \| \| (0.0) \| \| \| 0 \| \| \| (0.0) \| \| \| 0 \| \| \| (0.0) \| \| \| 0 \| \| \| (0.0) \| \| \| \|  \| \| \| \|  \| \| \|  \| \| \|  \| \| \|  \| \| \|  \| \| \|  \| \| \|  \| \| \|  \| \| \|  \| \| \|  \| \| \|  \| \| \|  \| \| \|  \| \| \|  \| \| \|  \| \| \|  \| \| \|  \| \| \| \| Nasopharynx \| \| \| \| 5 \| \| \| 1 \| \| \| (1.0) \| \| \| 24 \| \| \| (24.5) \| \| \| 5 \| \| \| (16.7) \| \| \| 92 \| \| \| (93.9) \| \| \| 1 \| \| \| (0.3) \| \| \| 33 \| \| \| (11.2) \| \| \| 13 \| \| \| (14.4) \| \| \| 249 \| \| \| (84.7) \| \| \| \|  \| \| \| \| 4 \| \| \| 11 \| \| \| (11.2) \| \| \| 66 \| \| \| (67.3) \| \| \| 25 \| \| \| (83.3) \| \| \| 6 \| \| \| (6.1) \| \| \| 34 \| \| \| (11.6) \| \| \| 196 \| \| \| (66.7) \| \| \| 75 \| \| \| (83.3) \| \| \| 44 \| \| \| (15.0) \| \| \| \|  \| \| \| \| 3 \| \| \| 85 \| \| \| (86.7) \| \| \| 8 \| \| \| (8.2) \| \| \| 0 \| \| \| (0.0) \| \| \| 0 \| \| \| (0.0) \| \| \| 257 \| \| \| (87.4) \| \| \| 63 \| \| \| (21.4) \| \| \| 2 \| \| \| (2.2) \| \| \| 1 \| \| \| (0.3) \| \| \| \|  \| \| \| \| 2 \| \| \| 1 \| \| \| (1.0) \| \| \| 0 \| \| \| (0.0) \| \| \| 0 \| \| \| (0.0) \| \| \| 0 \| \| \| (0.0) \| \| \| 2 \| \| \| (0.7) \| \| \| 2 \| \| \| (0.7) \| \| \| 0 \| \| \| (0.0) \| \| \| 0 \| \| \| (0.0) \| \| \| \|  \| \| \| \| 1 \| \| \| 0 \| \| \| (0.0) \| \| \| 0 \| \| \| (0.0) \| \| \| 0 \| \| \| (0.0) \| \| \| 0 \| \| \| (0.0) \| \| \| 0 \| \| \| (0.0) \| \| \| 0 \| \| \| (0.0) \| \| \| 0 \| \| \| (0.0) \| \| \| 0 \| \| \| (0.0) \| \| \| \|  \| \| \| \| \| \| \| **Reader 1** \| \| \| \| \| \| \| \| \| \| \| \| \| \| \| \| \| \| \| \| \| \| \| \| **Reader 2** \| \| \| \| \| \| \| \| \| \| \| \| \| \| \| \| \| \| \| \| \| \| \| \| \| **Parameter** \| \| \| \| \| \| \| **AIDR** \| \| \| \| \| \| **DL-1** \| \| \| \| \| \| **DL-1-SD** \| \| \| \| \| \| **DL-2** \| \| \| \| \| \| **AIDR** \| \| \| \| \| \| **DL-1** \| \| \| \| \| \| **DL-1-SD** \| \| \| \| \| \| **DL-2** \| \| \| \| \| \| \|  \| \| \| \| \| \| \| **n** \| \| \| **(%)** \| \| \| **n** \| \| \| **(%)** \| \| \| **n** \| \| \| **(%)** \| \| \| **n** \| \| \| **(%)** \| \| \| **n** \| \| \| **(%)** \| \| \| **n** \| \| \| **(%)** \| \| \| **n** \| \| \| **(%)** \| \| \| **n** \| \| \| **(%)** \| \| \| \| Hypopharynx \| \| \| \| 5 \| \| \| 0 \| \| \| (0.0) \| \| \| 0 \| \| \| (0.0) \| \| \| 6 \| \| \| (20.0) \| \| \| 80 \| \| \| (81.6) \| \| \| 0 \| \| \| (0.0) \| \| \| 9 \| \| \| (9.2) \| \| \| 0 \| \| \| (0.0) \| \| \| 73 \| \| \| (74.5) \| \| \| \| 4 \| \| \| 10 \| \| \| (10.2) \| \| \| 47 \| \| \| (48.0) \| \| \| 23 \| \| \| (76.7) \| \| \| 17 \| \| \| (17.3) \| \| \| 10 \| \| \| (10.2) \| \| \| 76 \| \| \| (77.6) \| \| \| 30 \| \| \| (100.0) \| \| \| 24 \| \| \| (24.5) \| \| \| \| 3 \| \| \| 88 \| \| \| (89.8) \| \| \| 50 \| \| \| (51.0) \| \| \| 1 \| \| \| (3.3) \| \| \| 1 \| \| \| (1.0) \| \| \| 87 \| \| \| (88.8) \| \| \| 13 \| \| \| (13.3) \| \| \| 0 \| \| \| (0.0) \| \| \| 1 \| \| \| (1.0) \| \| \| \| 2 \| \| \| 0 \| \| \| (0.0) \| \| \| 1 \| \| \| (1.0) \| \| \| 0 \| \| \| (0.0) \| \| \| 0 \| \| \| (0.0) \| \| \| 1 \| \| \| (1.0) \| \| \| 0 \| \| \| (0.0) \| \| \| 0 \| \| \| (0.0) \| \| \| 0 \| \| \| (0.0) \| \| \| \| 1 \| \| \| 0 \| \| \| (0.0) \| \| \| 0 \| \| \| (0.0) \| \| \| 0 \| \| \| (0.0) \| \| \| 0 \| \| \| (0.0) \| \| \| 0 \| \| \| (0.0) \| \| \| 0 \| \| \| (0.0) \| \| \| 0 \| \| \| (0.0) \| \| \| 0 \| \| \| (0.0) \| \| \| \|  \| \| \| \| \| \| \| \| \| \| \| \| \| \| \| \| \| \| \| \| \| \| \| \| \| \| \| \| \| \| \| \| \| \| \| \| \| \| \| \| \| \| \| \| \| \| \| \| \| \| \| \| \| \| \| \| Oral cavity \| \| \| \| 5 \| \| \| 0 \| \| \| (0.0) \| \| \| 0 \| \| \| (0.0) \| \| \| 2 \| \| \| (6.7) \| \| \| 35 \| \| \| (35.7) \| \| \| 0 \| \| \| (0.0) \| \| \| 5 \| \| \| (5.1) \| \| \| 0 \| \| \| (0.0) \| \| \| 29 \| \| \| (29.6) \| \| \| \| 4 \| \| \| 2 \| \| \| (2.0) \| \| \| 17 \| \| \| (17.3) \| \| \| 7 \| \| \| (23.3) \| \| \| 10 \| \| \| (10.2) \| \| \| 7 \| \| \| (7.1) \| \| \| 32 \| \| \| (32.7) \| \| \| 8 \| \| \| (26.7) \| \| \| 21 \| \| \| (21.4) \| \| \| \| 3 \| \| \| 52 \| \| \| (53.1) \| \| \| 51 \| \| \| (52.0) \| \| \| 11 \| \| \| (36.7) \| \| \| 30 \| \| \| (30.6) \| \| \| 65 \| \| \| (66.3) \| \| \| 40 \| \| \| (40.8) \| \| \| 12 \| \| \| (40.0) \| \| \| 17 \| \| \| (17.3) \| \| \| \| 2 \| \| \| 44 \| \| \| (44.9) \| \| \| 30 \| \| \| (30.6) \| \| \| 10 \| \| \| (33.3) \| \| \| 23 \| \| \| (23.5) \| \| \| 26 \| \| \| (26.5) \| \| \| 21 \| \| \| (21.4) \| \| \| 10 \| \| \| (33.3) \| \| \| 31 \| \| \| (31.6) \| \| \| \| 1 \| \| \| 0 \| \| \| (0.0) \| \| \| 0 \| \| \| (0.0) \| \| \| 0 \| \| \| (0.0) \| \| \| 0 \| \| \| (0.0) \| \| \| 0 \| \| \| (0.0) \| \| \| 0 \| \| \| (0.0) \| \| \| 0 \| \| \| (0.0) \| \| \| 0 \| \| \| (0.0) \| \| \| \|  \| \| \| \| \| \| \| \| \| \| \| \| \| \| \| \| \| \| \| \| \| \| \| \| \| \| \| \| \| \| \| \| \| \| \| \| \| \| \| \| \| \| \| \| \| \| \| \| \| \| \| \| \| \| \| \| Floor of mouth \| \| \| \| 5 \| \| \| 0 \| \| \| (0.0) \| \| \| 0 \| \| \| (0.0) \| \| \| 5 \| \| \| (16.7) \| \| \| 72 \| \| \| (73.5) \| \| \| 0 \| \| \| (0.0) \| \| \| 10 \| \| \| (10.2) \| \| \| 0 \| \| \| (0.0) \| \| \| 60 \| \| \| (61.2) \| \| \| \| 4 \| \| \| 6 \| \| \| (6.1) \| \| \| 38 \| \| \| (38.8) \| \| \| 23 \| \| \| (76.7) \| \| \| 21 \| \| \| (21.4) \| \| \| 13 \| \| \| (13.3) \| \| \| 67 \| \| \| (68.4) \| \| \| 28 \| \| \| (93.3) \| \| \| 33 \| \| \| (33.7) \| \| \| \|  \| \| \| \| 3 \| \| \| 88 \| \| \| (89.8) \| \| \| 58 \| \| \| (59.2) \| \| \| 2 \| \| \| (6.7) \| \| \| 4 \| \| \| (4.1) \| \| \| 82 \| \| \| (83.7) \| \| \| 21 \| \| \| (21.4) \| \| \| 2 \| \| \| (6.7) \| \| \| 5 \| \| \| (5.1) \| \| \| \|  \| \| \| \| 2 \| \| \| 4 \| \| \| (4.1) \| \| \| 2 \| \| \| (2.0) \| \| \| 0 \| \| \| (0.0) \| \| \| 1 \| \| \| (1.0) \| \| \| 3 \| \| \| (3.1) \| \| \| 0 \| \| \| (0.0) \| \| \| 0 \| \| \| (0.0) \| \| \| 0 \| \| \| (0.0) \| \| \| \|  \| \| \| \| 1 \| \| \| 0 \| \| \| (0.0) \| \| \| 0 \| \| \| (0.0) \| \| \| 0 \| \| \| (0.0) \| \| \| 0 \| \| \| (0.0) \| \| \| 0 \| \| \| (0.0) \| \| \| 0 \| \| \| (0.0) \| \| \| 0 \| \| \| (0.0) \| \| \| 0 \| \| \| (0.0) \| \| \| \|  \| \| \| \|  \| \| \|  \| \| \|  \| \| \|  \| \| \|  \| \| \|  \| \| \|  \| \| \|  \| \| \|  \| \| \|  \| \| \|  \| \| \|  \| \| \|  \| \| \|  \| \| \|  \| \| \|  \| \| \|  \| \| \| \| Salivary glands \| \| \| \| 5 \| \| \| 0 \| \| \| (0.0) \| \| \| 0 \| \| \| (0.0) \| \| \| 7 \| \| \| (23.3) \| \| \| 69 \| \| \| (70.4) \| \| \| 0 \| \| \| (0.0) \| \| \| 10 \| \| \| (10.2) \| \| \| 0 \| \| \| (0.0) \| \| \| 63 \| \| \| (64.3) \| \| \| \|  \| \| \| \| 4 \| \| \| 9 \| \| \| (9.2) \| \| \| 40 \| \| \| (40.8) \| \| \| 22 \| \| \| (73.3) \| \| \| 23 \| \| \| (23.5) \| \| \| 7 \| \| \| (7.1) \| \| \| 71 \| \| \| (72.4) \| \| \| 30 \| \| \| (100.0) \| \| \| 33 \| \| \| (33.7) \| \| \| \|  \| \| \| \| 3 \| \| \| 88 \| \| \| (89.8) \| \| \| 57 \| \| \| (58.2) \| \| \| 1 \| \| \| (3.3) \| \| \| 5 \| \| \| (5.1) \| \| \| 90 \| \| \| (91.8) \| \| \| 17 \| \| \| (17.3) \| \| \| 0 \| \| \| (0.0) \| \| \| 2 \| \| \| (2.0) \| \| \| \|  \| \| \| \| 2 \| \| \| 1 \| \| \| (1.0) \| \| \| 1 \| \| \| (1.0) \| \| \| 0 \| \| \| (0.0) \| \| \| 1 \| \| \| (1.0) \| \| \| 1 \| \| \| (1.0) \| \| \| 0 \| \| \| (0.0) \| \| \| 0 \| \| \| (0.0) \| \| \| 0 \| \| \| (0.0) \| \| \| \|  \| \| \| \| 1 \| \| \| 0 \| \| \| (0.0) \| \| \| 0 \| \| \| (0.0) \| \| \| 0 \| \| \| (0.0) \| \| \| 0 \| \| \| (0.0) \| \| \| 0 \| \| \| (0.0) \| \| \| 0 \| \| \| (0.0) \| \| \| 0 \| \| \| (0.0) \| \| \| 0 \| \| \| (0.0) \| \| \| \|  \| \| \| \|  \| \| \|  \| \| \|  \| \| \|  \| \| \|  \| \| \|  \| \| \|  \| \| \|  \| \| \|  \| \| \|  \| \| \|  \| \| \|  \| \| \|  \| \| \|  \| \| \|  \| \| \|  \| \| \|  \| \| \| \| Lymphnodes \| \| \| \| 5 \| \| \| 0 \| \| \| (0.0) \| \| \| 0 \| \| \| (0.0) \| \| \| 6 \| \| \| (20.0) \| \| \| 64 \| \| \| (65.3) \| \| \| 0 \| \| \| (0.0) \| \| \| 10 \| \| \| (10.2) \| \| \| 0 \| \| \| (0.0) \| \| \| 61 \| \| \| (62.2) \| \| \| \|  \| \| \| \| 4 \| \| \| 11 \| \| \| (11.2) \| \| \| 49 \| \| \| (50.0) \| \| \| 24 \| \| \| (80.0) \| \| \| 33 \| \| \| (33.7) \| \| \| 10 \| \| \| (10.2) \| \| \| 75 \| \| \| (76.5) \| \| \| 30 \| \| \| (100.0) \| \| \| 37 \| \| \| (37.8) \| \| \| \|  \| \| \| \| 3 \| \| \| 87 \| \| \| (88.8) \| \| \| 49 \| \| \| (50.0) \| \| \| 0 \| \| \| (0.0) \| \| \| 1 \| \| \| (1.0) \| \| \| 87 \| \| \| (88.8) \| \| \| 13 \| \| \| (13.3) \| \| \| 0 \| \| \| (0.0) \| \| \| 0 \| \| \| (0.0) \| \| \| \|  \| \| \| \| 2 \| \| \| 0 \| \| \| (0.0) \| \| \| 0 \| \| \| (0.0) \| \| \| 0 \| \| \| (0.0) \| \| \| 0 \| \| \| (0.0) \| \| \| 1 \| \| \| (1.0) \| \| \| 0 \| \| \| (0.0) \| \| \| 0 \| \| \| (0.0) \| \| \| 0 \| \| \| (0.0) \| \| \| \|  \| \| \| \| 1 \| \| \| 0 \| \| \| (0.0) \| \| \| 0 \| \| \| (0.0) \| \| \| 0 \| \| \| (0.0) \| \| \| 0 \| \| \| (0.0) \| \| \| 0 \| \| \| (0.0) \| \| \| 0 \| \| \| (0.0) \| \| \| 0 \| \| \| (0.0) \| \| \| 0 \| \| \| (0.0) \| \| \| \|  \| \| \| \|  \| \| \|  \| \| \|  \| \| \|  \| \| \|  \| \| \|  \| \| \|  \| \| \|  \| \| \|  \| \| \|  \| \| \|  \| \| \|  \| \| \|  \| \| \|  \| \| \|  \| \| \|  \| \| \|  \| \| \| \| Pterygopalatine fossa \| \| \| \| 5 \| \| \| 0 \| \| \| (0.0) \| \| \| 0 \| \| \| (0.0) \| \| \| 7 \| \| \| (23.3) \| \| \| 80 \| \| \| (81.6) \| \| \| 0 \| \| \| (0.0) \| \| \| 10 \| \| \| (10.2) \| \| \| 0 \| \| \| (0.0) \| \| \| 73 \| \| \| (74.5) \| \| \| \| 4 \| \| \| 13 \| \| \| (13.3) \| \| \| 45 \| \| \| (45.9) \| \| \| 23 \| \| \| (76.7) \| \| \| 18 \| \| \| (18.4) \| \| \| 12 \| \| \| (12.2) \| \| \| 82 \| \| \| (83.7) \| \| \| 30 \| \| \| (100.0) \| \| \| 25 \| \| \| (25.5) \| \| \| \|  \| \| \| \| 3 \| \| \| 85 \| \| \| (86.7) \| \| \| 53 \| \| \| (54.1) \| \| \| 0 \| \| \| (0.0) \| \| \| 0 \| \| \| (0.0) \| \| \| 86 \| \| \| (87.8) \| \| \| 6 \| \| \| (6.1) \| \| \| 0 \| \| \| (0.0) \| \| \| 0 \| \| \| (0.0) \| \| \| \|  \| \| \| \| 2 \| \| \| 0 \| \| \| (0.0) \| \| \| 0 \| \| \| (0.0) \| \| \| 0 \| \| \| (0.0) \| \| \| 0 \| \| \| (0.0) \| \| \| 0 \| \| \| (0.0) \| \| \| 0 \| \| \| (0.0) \| \| \| 0 \| \| \| (0.0) \| \| \| 0 \| \| \| (0.0) \| \| \| \|  \| \| \| \| 1 \| \| \| 0 \| \| \| (0.0) \| \| \| 0 \| \| \| (0.0) \| \| \| 0 \| \| \| (0.0) \| \| \| 0 \| \| \| (0.0) \| \| \| 0 \| \| \| (0.0) \| \| \| 0 \| \| \| (0.0) \| \| \| 0 \| \| \| (0.0) \| \| \| 0 \| \| \| (0.0) \| \| \| \|  \| \| \| \|  \| \| \|  \| \| \|  \| \| \|  \| \| \|  \| \| \|  \| \| \|  \| \| \|  \| \| \|  \| \| \|  \| \| \|  \| \| \|  \| \| \|  \| \| \|  \| \| \|  \| \| \|  \| \| \|  \| \| \| \|  \| \| \| \| \| \| \| **Reader 3** \| \| \| \| \| \| \| \| \| \| \| \| \| \| \| \| \| \| \| \| \| \| \| \| **Overall** \| \| \| \| \| \| \| \| \| \| \| \| \| \| \| \| \| \| \| \| \| \| \| \| \| **Parameter** \| \| \| \| \| \| \| **AIDR** \| \| \| \| \| \| **DL-1** \| \| \| \| \| \| **DL-1-SD** \| \| \| \| \| \| **DL-2** \| \| \| \| \| \| **AIDR** \| \| \| \| \| \| **DL-1** \| \| \| \| \| \| **DL-1-SD** \| \| \| \| \| \| **DL-2** \| \| \| \| \| \| \|  \| \| \| \| \| \| \| **n** \| \| \| **(%)** \| \| \| **n** \| \| \| **(%)** \| \| \| **n** \| \| \| **(%)** \| \| \| **n** \| \| \| **(%)** \| \| \| **n** \| \| \| **(%)** \| \| \| **n** \| \| \| **(%)** \| \| \| **n** \| \| \| **(%)** \| \| \| **n** \| \| \| **(%)** \| \| \| \| Hypopharynx \| \| \| \| 5 \| \| \| 1 \| \| \| (1.0) \| \| \| 20 \| \| \| (20.4) \| \| \| 4 \| \| \| (13.3) \| \| \| 88 \| \| \| (89.8) \| \| \| 1 \| \| \| (0.3) \| \| \| 29 \| \| \| (9.9) \| \| \| 10 \| \| \| (11.1) \| \| \| 241 \| \| \| (82.0) \| \| \| \|  \| \| \| \| 4 \| \| \| 12 \| \| \| (12.2) \| \| \| 68 \| \| \| (69.4) \| \| \| 26 \| \| \| (86.7) \| \| \| 8 \| \| \| (8.2) \| \| \| 32 \| \| \| (10.9) \| \| \| 191 \| \| \| (65.0) \| \| \| 79 \| \| \| (87.8) \| \| \| 49 \| \| \| (16.7) \| \| \| \|  \| \| \| \| 3 \| \| \| 82 \| \| \| (83.7) \| \| \| 10 \| \| \| (10.2) \| \| \| 0 \| \| \| (0.0) \| \| \| 2 \| \| \| (2.0) \| \| \| 257 \| \| \| (87.4) \| \| \| 73 \| \| \| (24.8) \| \| \| 1 \| \| \| (1.1) \| \| \| 4 \| \| \| (1.4) \| \| \| \|  \| \| \| \| 2 \| \| \| 3 \| \| \| (3.1) \| \| \| 0 \| \| \| (0.0) \| \| \| 0 \| \| \| (0.0) \| \| \| 0 \| \| \| (0.0) \| \| \| 4 \| \| \| (1.4) \| \| \| 1 \| \| \| (0.3) \| \| \| 0 \| \| \| (0.0) \| \| \| 0 \| \| \| (0.0) \| \| \| \|  \| \| \| \| 1 \| \| \| 0 \| \| \| (0.0) \| \| \| 0 \| \| \| (0.0) \| \| \| 0 \| \| \| (0.0) \| \| \| 0 \| \| \| (0.0) \| \| \| 0 \| \| \| (0.0) \| \| \| 0 \| \| \| (0.0) \| \| \| 0 \| \| \| (0.0) \| \| \| 0 \| \| \| (0.0) \| \| \| \|  \| \| \| \|  \| \| \|  \| \| \|  \| \| \|  \| \| \|  \| \| \|  \| \| \|  \| \| \|  \| \| \|  \| \| \|  \| \| \|  \| \| \|  \| \| \|  \| \| \|  \| \| \|  \| \| \|  \| \| \|  \| \| \| \| Oral cavity \| \| \| \| 5 \| \| \| 1 \| \| \| (1.0) \| \| \| 13 \| \| \| (13.3) \| \| \| 0 \| \| \| (0.0) \| \| \| 41 \| \| \| (41.8) \| \| \| 1 \| \| \| (0.3) \| \| \| 18 \| \| \| (6.1) \| \| \| 2 \| \| \| (2.2) \| \| \| 105 \| \| \| (35.7) \| \| \| \| 4 \| \| \| 11 \| \| \| (11.2) \| \| \| 37 \| \| \| (37.8) \| \| \| 25 \| \| \| (83.3) \| \| \| 24 \| \| \| (24.5) \| \| \| 20 \| \| \| (6.8) \| \| \| 86 \| \| \| (29.3) \| \| \| 40 \| \| \| (44.4) \| \| \| 55 \| \| \| (18.7) \| \| \| \| 3 \| \| \| 75 \| \| \| (76.5) \| \| \| 36 \| \| \| (36.7) \| \| \| 5 \| \| \| (16.7) \| \| \| 31 \| \| \| (31.6) \| \| \| 192 \| \| \| (65.3) \| \| \| 127 \| \| \| (43.2) \| \| \| 28 \| \| \| (31.1) \| \| \| 78 \| \| \| (26.5) \| \| \| \| 2 \| \| \| 11 \| \| \| (11.2) \| \| \| 12 \| \| \| (12.2) \| \| \| 0 \| \| \| (0.0) \| \| \| 2 \| \| \| (2.0) \| \| \| 81 \| \| \| (27.6) \| \| \| 63 \| \| \| (21.4) \| \| \| 20 \| \| \| (22.2) \| \| \| 56 \| \| \| (19.0) \| \| \| \| 1 \| \| \| 0 \| \| \| (0.0) \| \| \| 0 \| \| \| (0.0) \| \| \| 0 \| \| \| (0.0) \| \| \| 0 \| \| \| (0.0) \| \| \| 0 \| \| \| (0.0) \| \| \| 0 \| \| \| (0.0) \| \| \| 0 \| \| \| (0.0) \| \| \| 0 \| \| \| (0.0) \| \| \| \|  \| \| \| \| \| \| \| \| \| \| \| \| \| \| \| \| \| \| \| \| \| \| \| \| \| \| \| \| \| \| \| \| \| \| \| \| \| \| \| \| \| \| \| \| \| \| \| \| \| \| \| \| \| \| \| \| Floor of mouth \| \| \| \| 5 \| \| \| 1 \| \| \| (1.0) \| \| \| 22 \| \| \| (22.4) \| \| \| 1 \| \| \| (3.3) \| \| \| 80 \| \| \| (81.6) \| \| \| 1 \| \| \| (0.3) \| \| \| 32 \| \| \| (10.9) \| \| \| 6 \| \| \| (6.7) \| \| \| 212 \| \| \| (72.1) \| \| \| \| 4 \| \| \| 13 \| \| \| (13.3) \| \| \| 58 \| \| \| (59.2) \| \| \| 24 \| \| \| (80.0) \| \| \| 17 \| \| \| (17.3) \| \| \| 32 \| \| \| (10.9) \| \| \| 163 \| \| \| (55.4) \| \| \| 75 \| \| \| (83.3) \| \| \| 71 \| \| \| (24.1) \| \| \| \| 3 \| \| \| 83 \| \| \| (84.7) \| \| \| 17 \| \| \| (17.3) \| \| \| 5 \| \| \| (16.7) \| \| \| 1 \| \| \| (1.0) \| \| \| 253 \| \| \| (86.1) \| \| \| 96 \| \| \| (32.7) \| \| \| 9 \| \| \| (10.0) \| \| \| 10 \| \| \| (3.4) \| \| \| \| 2 \| \| \| 1 \| \| \| (1.0) \| \| \| 1 \| \| \| (1.0) \| \| \| 0 \| \| \| (0.0) \| \| \| 0 \| \| \| (0.0) \| \| \| 8 \| \| \| (2.7) \| \| \| 3 \| \| \| (1.0) \| \| \| 0 \| \| \| (0.0) \| \| \| 1 \| \| \| (0.3) \| \| \| \| 1 \| \| \| 0 \| \| \| (0.0) \| \| \| 0 \| \| \| (0.0) \| \| \| 0 \| \| \| (0.0) \| \| \| 0 \| \| \| (0.0) \| \| \| 0 \| \| \| (0.0) \| \| \| 0 \| \| \| (0.0) \| \| \| 0 \| \| \| (0.0) \| \| \| 0 \| \| \| (0.0) \| \| \| \|  \| \| \| \| \| \| \| \| \| \| \| \| \| \| \| \| \| \| \| \| \| \| \| \| \| \| \| \| \| \| \| \| \| \| \| \| \| \| \| \| \| \| \| \| \| \| \| \| \| \| \| \| \| \| \| \| Salivary glands \| \| \| \| 5 \| \| \| 1 \| \| \| (1.0) \| \| \| 22 \| \| \| (22.4) \| \| \| 5 \| \| \| (16.7) \| \| \| 82 \| \| \| (83.7) \| \| \| 1 \| \| \| (0.3) \| \| \| 32 \| \| \| (10.9) \| \| \| 12 \| \| \| (13.3) \| \| \| 214 \| \| \| (72.8) \| \| \| \| 4 \| \| \| 16 \| \| \| (16.3) \| \| \| 62 \| \| \| (63.3) \| \| \| 25 \| \| \| (83.3) \| \| \| 15 \| \| \| (15.3) \| \| \| 32 \| \| \| (10.9) \| \| \| 173 \| \| \| (58.8) \| \| \| 77 \| \| \| (85.6) \| \| \| 71 \| \| \| (24.1) \| \| \| \| 3 \| \| \| 80 \| \| \| (81.6) \| \| \| 13 \| \| \| (13.3) \| \| \| 0 \| \| \| (0.0) \| \| \| 1 \| \| \| (1.0) \| \| \| 258 \| \| \| (87.8) \| \| \| 87 \| \| \| (29.6) \| \| \| 1 \| \| \| (1.1) \| \| \| 8 \| \| \| (2.7) \| \| \| \|  \| \| \| \| 2 \| \| \| 1 \| \| \| (1.0) \| \| \| 1 \| \| \| (1.0) \| \| \| 0 \| \| \| (0.0) \| \| \| 0 \| \| \| (0.0) \| \| \| 3 \| \| \| (1.0) \| \| \| 2 \| \| \| (0.7) \| \| \| 0 \| \| \| (0.0) \| \| \| 1 \| \| \| (0.3) \| \| \| \|  \| \| \| \| 1 \| \| \| 0 \| \| \| (0.0) \| \| \| 0 \| \| \| (0.0) \| \| \| 0 \| \| \| (0.0) \| \| \| 0 \| \| \| (0.0) \| \| \| 0 \| \| \| (0.0) \| \| \| 0 \| \| \| (0.0) \| \| \| 0 \| \| \| (0.0) \| \| \| 0 \| \| \| (0.0) \| \| \| \|  \| \| \| \|  \| \| \|  \| \| \|  \| \| \|  \| \| \|  \| \| \|  \| \| \|  \| \| \|  \| \| \|  \| \| \|  \| \| \|  \| \| \|  \| \| \|  \| \| \|  \| \| \|  \| \| \|  \| \| \|  \| \| \| \| Lymphnodes \| \| \| \| 5 \| \| \| 1 \| \| \| (1.0) \| \| \| 28 \| \| \| (28.6) \| \| \| 10 \| \| \| (33.3) \| \| \| 82 \| \| \| (83.7) \| \| \| 1 \| \| \| (0.3) \| \| \| 38 \| \| \| (12.9) \| \| \| 16 \| \| \| (17.8) \| \| \| 207 \| \| \| (70.4) \| \| \| \|  \| \| \| \| 4 \| \| \| 21 \| \| \| (21.4) \| \| \| 59 \| \| \| (60.2) \| \| \| 20 \| \| \| (66.7) \| \| \| 14 \| \| \| (14.3) \| \| \| 42 \| \| \| (14.3) \| \| \| 183 \| \| \| (62.2) \| \| \| 74 \| \| \| (82.2) \| \| \| 84 \| \| \| (28.6) \| \| \| \|  \| \| \| \| 3 \| \| \| 74 \| \| \| (75.5) \| \| \| 11 \| \| \| (11.2) \| \| \| 0 \| \| \| (0.0) \| \| \| 2 \| \| \| (2.0) \| \| \| 248 \| \| \| (84.4) \| \| \| 73 \| \| \| (24.8) \| \| \| 0 \| \| \| (0.0) \| \| \| 3 \| \| \| (1.0) \| \| \| \|  \| \| \| \| 2 \| \| \| 2 \| \| \| (2.0) \| \| \| 0 \| \| \| (0.0) \| \| \| 0 \| \| \| (0.0) \| \| \| 0 \| \| \| (0.0) \| \| \| 3 \| \| \| (1.0) \| \| \| 0 \| \| \| (0.0) \| \| \| 0 \| \| \| (0.0) \| \| \| 0 \| \| \| (0.0) \| \| \| \|  \| \| \| \| 1 \| \| \| 0 \| \| \| (0.0) \| \| \| 0 \| \| \| (0.0) \| \| \| 0 \| \| \| (0.0) \| \| \| 0 \| \| \| (0.0) \| \| \| 0 \| \| \| (0.0) \| \| \| 0 \| \| \| (0.0) \| \| \| 0 \| \| \| ( 0.0) \| \| \| 0 \| \| \| (0.0) \| \| \| \|  \| \| \| \|  \| \| \|  \| \| \|  \| \| \|  \| \| \|  \| \| \|  \| \| \|  \| \| \|  \| \| \|  \| \| \|  \| \| \|  \| \| \|  \| \| \|  \| \| \|  \| \| \|  \| \| \|  \| \| \|  \| \| \| \| Pterygopalatine fossa \| \| \| \| 5 \| \| \| 1 \| \| \| (1.0) \| \| \| 21 \| \| \| (21.4) \| \| \| 6 \| \| \| (20.0) \| \| \| 89 \| \| \| (90.8) \| \| \| 1 \| \| \| (0.3) \| \| \| 31 \| \| \| (10.5) \| \| \| 13 \| \| \| (14.4) \| \| \| 242 \| \| \| (82.3) \| \| \| \| 4 \| \| \| 16 \| \| \| (16.3) \| \| \| 71 \| \| \| (72.4) \| \| \| 24 \| \| \| (80.0) \| \| \| 9 \| \| \| (9.2) \| \| \| 41 \| \| \| (13.9) \| \| \| 198 \| \| \| (67.3) \| \| \| 77 \| \| \| (85.6) \| \| \| 52 \| \| \| (17.7) \| \| \| \|  \| \| \| \| 3 \| \| \| 81 \| \| \| (82.7) \| \| \| 6 \| \| \| (6.1) \| \| \| 0 \| \| \| (0.0) \| \| \| 0 \| \| \| (0.0) \| \| \| 252 \| \| \| (85.7) \| \| \| 65 \| \| \| (22.1) \| \| \| 0 \| \| \| (0.0) \| \| \| 0 \| \| \| (0.0) \| \| \| \| \| \|  \| \| \| \| 2 \| \| \| 0 \| \| \| (0.0) \| \| \| 0 \| \| \| (0.0) \| \| \| 0 \| \| \| (0.0) \| \| \| 0 \| \| \| (0.0) \| \| \| 0 \| \| \| (0.0) \| \| \| 0 \| \| \| (0.0) \| \| \| 0 \| \| \| (0.0) \| \| \| 0 \| \| \| (0.0) \| \| \| \| \| \|  \| \| \| \| 1 \| \| \| 0 \| \| \| (0.0) \| \| \| 0 \| \| \| (0.0) \| \| \| 0 \| \| \| (0.0) \| \| \| 0 \| \| \| (0.0) \| \| \| 0 \| \| \| (0.0) \| \| \| 0 \| \| \| (0.0) \| \| \| 0 \| \| \| (0.0) \| \| \| 0 \| \| \| (0.0) \| \| \| \| \| \| n: Number of observations; (%): Percentage based on number of non-missing observations; Adaptive Iterative Dose Reduction (AIDR); Deep-learning Reconstruction with lower dose (DL-1); Deep-learning Reconstruction with standard dose (DL-1-SD); Novel Deep-learning Reconstruction with lower dose (DL-2) \| \| \| \| \| \| \| \| \| \| \| \| \| \| \| \| \| \| \| \| \| \| \| \| \| \| \| \| \| \| \| \| \| \| \| \| \| \| \| \| \| \| \| \| \| \| \| \| \| \| \| \| \| \| \| \| \|   **Table S4 Odds Ratios for different techniques** | | | | | | | | | |  |
| --- | --- | --- | --- | --- | --- | --- | --- | --- | --- | --- | --- | --- | --- | --- | --- | --- | --- | --- | --- | --- | --- | --- | --- | --- | --- | --- | --- | --- | --- | --- | --- | --- | --- | --- | --- | --- | --- | --- | --- | --- | --- | --- | --- | --- | --- | --- | --- | --- | --- | --- | --- | --- | --- | --- | --- | --- | --- | --- | --- | --- | --- | --- | --- | --- | --- | --- | --- | --- | --- | --- | --- | --- | --- | --- | --- | --- | --- | --- | --- | --- | --- | --- | --- | --- | --- | --- | --- | --- | --- | --- | --- | --- | --- | --- | --- | --- | --- | --- | --- | --- | --- | --- | --- | --- | --- | --- | --- | --- | --- | --- | --- | --- | --- | --- | --- | --- | --- | --- | --- | --- | --- | --- | --- | --- | --- | --- | --- | --- | --- | --- | --- | --- | --- | --- | --- | --- | --- | --- | --- | --- | --- | --- | --- | --- | --- | --- | --- | --- | --- | --- | --- | --- | --- | --- | --- | --- | --- | --- | --- | --- | --- | --- | --- | --- | --- | --- | --- | --- | --- | --- | --- | --- | --- | --- | --- | --- | --- | --- | --- | --- | --- | --- | --- | --- | --- | --- | --- | --- | --- | --- | --- | --- | --- | --- | --- | --- | --- | --- | --- | --- | --- | --- | --- | --- | --- | --- | --- | --- | --- | --- | --- | --- | --- | --- | --- | --- | --- | --- | --- | --- | --- | --- | --- | --- | --- | --- | --- | --- | --- | --- | --- | --- | --- | --- | --- | --- | --- | --- | --- | --- | --- | --- | --- | --- | --- | --- | --- | --- | --- | --- | --- | --- | --- | --- | --- | --- | --- | --- | --- | --- | --- | --- | --- | --- | --- | --- | --- | --- | --- | --- | --- | --- | --- | --- | --- | --- | --- | --- | --- | --- | --- | --- | --- | --- | --- | --- | --- | --- | --- | --- | --- | --- | --- | --- | --- | --- | --- | --- | --- | --- | --- | --- | --- | --- | --- | --- | --- | --- | --- | --- | --- | --- | --- | --- | --- | --- | --- | --- | --- | --- | --- | --- | --- | --- | --- | --- | --- | --- | --- | --- | --- | --- | --- | --- | --- | --- | --- | --- | --- | --- | --- | --- | --- | --- | --- | --- | --- | --- | --- | --- | --- | --- | --- | --- | --- | --- | --- | --- | --- | --- | --- | --- | --- | --- | --- | --- | --- | --- | --- | --- | --- | --- | --- | --- | --- | --- | --- | --- | --- | --- | --- | --- | --- | --- | --- | --- | --- | --- | --- | --- | --- | --- | --- | --- | --- | --- | --- | --- | --- | --- | --- | --- | --- | --- | --- | --- | --- | --- | --- | --- | --- | --- | --- | --- | --- | --- | --- | --- | --- | --- | --- | --- | --- | --- | --- | --- | --- | --- | --- | --- | --- | --- | --- | --- | --- | --- | --- | --- | --- | --- | --- | --- | --- | --- | --- | --- | --- | --- | --- | --- | --- | --- | --- | --- | --- | --- | --- | --- | --- | --- | --- | --- | --- | --- | --- | --- | --- | --- | --- | --- | --- | --- | --- | --- | --- | --- | --- | --- | --- | --- | --- | --- | --- | --- | --- | --- | --- | --- | --- | --- | --- | --- | --- | --- | --- | --- | --- | --- | --- | --- | --- | --- | --- | --- | --- | --- | --- | --- | --- | --- | --- | --- | --- | --- | --- | --- | --- | --- | --- | --- | --- | --- | --- | --- | --- | --- | --- | --- | --- | --- | --- | --- | --- | --- | --- | --- | --- | --- | --- | --- | --- | --- | --- | --- | --- | --- | --- | --- | --- | --- | --- | --- | --- | --- | --- | --- | --- | --- | --- | --- | --- | --- | --- | --- | --- | --- | --- | --- | --- | --- | --- | --- | --- | --- | --- | --- | --- | --- | --- | --- | --- | --- | --- | --- | --- | --- | --- | --- | --- | --- | --- | --- | --- | --- | --- | --- | --- | --- | --- | --- | --- | --- | --- | --- | --- | --- | --- | --- | --- | --- | --- | --- | --- | --- | --- | --- | --- | --- | --- | --- | --- | --- | --- | --- | --- | --- | --- | --- | --- | --- | --- | --- | --- | --- | --- | --- | --- | --- | --- | --- | --- | --- | --- | --- | --- | --- | --- | --- | --- | --- | --- | --- | --- | --- | --- | --- | --- | --- | --- | --- | --- | --- | --- | --- | --- | --- | --- | --- | --- | --- | --- | --- | --- | --- | --- | --- | --- | --- | --- | --- | --- | --- | --- | --- | --- | --- | --- | --- | --- | --- | --- | --- | --- | --- | --- | --- | --- | --- | --- | --- | --- | --- | --- | --- | --- | --- | --- | --- | --- | --- | --- | --- | --- | --- | --- | --- | --- | --- | --- | --- | --- | --- | --- | --- | --- | --- | --- | --- | --- | --- | --- | --- | --- | --- | --- | --- | --- | --- | --- | --- | --- | --- | --- | --- | --- | --- | --- | --- | --- | --- | --- | --- | --- | --- | --- | --- | --- | --- | --- | --- | --- | --- | --- | --- | --- | --- | --- | --- | --- | --- | --- | --- | --- | --- | --- | --- | --- | --- | --- | --- | --- | --- | --- | --- | --- | --- | --- | --- | --- | --- | --- | --- | --- | --- | --- | --- | --- | --- | --- | --- | --- | --- | --- | --- | --- | --- | --- | --- | --- | --- | --- | --- | --- | --- | --- | --- | --- | --- | --- | --- | --- | --- | --- | --- | --- | --- | --- | --- | --- | --- | --- | --- | --- | --- | --- | --- | --- | --- | --- | --- | --- | --- | --- | --- | --- | --- | --- | --- | --- | --- | --- | --- | --- | --- | --- | --- | --- | --- | --- | --- | --- | --- | --- | --- | --- | --- | --- | --- | --- | --- | --- | --- | --- | --- | --- | --- | --- | --- | --- | --- | --- | --- | --- | --- | --- | --- | --- | --- | --- | --- | --- | --- | --- | --- | --- | --- | --- | --- | --- | --- | --- | --- | --- | --- | --- | --- | --- | --- | --- | --- | --- | --- | --- | --- | --- | --- | --- | --- | --- | --- | --- | --- | --- | --- | --- | --- | --- | --- | --- | --- | --- | --- | --- | --- | --- | --- | --- | --- | --- | --- | --- | --- | --- | --- | --- | --- | --- | --- | --- | --- | --- | --- | --- | --- | --- | --- | --- | --- | --- | --- | --- | --- | --- | --- | --- | --- | --- | --- | --- | --- | --- | --- | --- | --- | --- | --- | --- | --- | --- | --- | --- | --- | --- | --- | --- | --- | --- | --- | --- | --- | --- | --- | --- | --- | --- | --- | --- | --- | --- | --- | --- | --- | --- | --- | --- | --- | --- | --- | --- | --- | --- | --- | --- | --- | --- | --- | --- | --- | --- | --- | --- | --- | --- | --- | --- | --- | --- | --- | --- | --- | --- | --- | --- | --- | --- | --- | --- | --- | --- | --- | --- | --- | --- | --- | --- | --- | --- | --- | --- | --- | --- | --- | --- | --- | --- | --- | --- | --- | --- | --- | --- | --- | --- | --- | --- | --- | --- | --- | --- | --- | --- | --- | --- | --- | --- | --- | --- | --- | --- | --- | --- | --- | --- | --- | --- | --- | --- | --- | --- | --- | --- | --- | --- | --- | --- | --- | --- | --- | --- | --- | --- | --- | --- | --- | --- | --- | --- | --- | --- | --- | --- | --- | --- | --- | --- | --- | --- | --- | --- | --- | --- | --- | --- | --- | --- | --- | --- | --- | --- | --- | --- | --- | --- | --- | --- | --- | --- | --- | --- | --- | --- | --- | --- | --- | --- | --- | --- | --- | --- | --- | --- | --- | --- | --- | --- | --- | --- | --- | --- | --- | --- | --- | --- | --- | --- | --- | --- | --- | --- | --- | --- | --- | --- | --- | --- | --- | --- | --- | --- | --- | --- | --- | --- | --- | --- | --- | --- | --- | --- | --- | --- | --- | --- | --- | --- | --- | --- | --- | --- | --- | --- | --- | --- | --- | --- | --- | --- | --- | --- | --- | --- | --- | --- | --- | --- | --- | --- | --- | --- | --- | --- | --- | --- | --- | --- | --- | --- | --- | --- | --- | --- | --- | --- | --- | --- | --- | --- | --- | --- | --- | --- | --- | --- | --- | --- | --- | --- | --- | --- | --- | --- | --- | --- | --- | --- | --- | --- | --- | --- | --- | --- | --- | --- | --- | --- | --- | --- | --- | --- | --- | --- | --- | --- | --- | --- | --- | --- | --- | --- | --- | --- | --- | --- | --- | --- | --- | --- | --- | --- | --- | --- | --- | --- | --- | --- | --- | --- | --- | --- | --- | --- | --- | --- | --- | --- | --- | --- | --- | --- | --- | --- | --- | --- | --- | --- | --- | --- | --- | --- | --- | --- | --- | --- | --- | --- | --- | --- | --- | --- | --- | --- | --- | --- | --- | --- | --- | --- | --- | --- | --- | --- | --- | --- | --- | --- | --- | --- | --- | --- | --- | --- | --- | --- | --- | --- | --- | --- | --- | --- | --- | --- | --- | --- | --- | --- | --- | --- | --- | --- | --- | --- | --- | --- | --- | --- | --- | --- | --- | --- | --- | --- | --- | --- | --- | --- | --- | --- | --- | --- | --- | --- | --- | --- | --- | --- | --- | --- | --- | --- | --- | --- | --- | --- | --- | --- | --- | --- | --- | --- | --- | --- | --- | --- | --- | --- | --- | --- | --- | --- | --- | --- | --- | --- | --- | --- | --- | --- | --- | --- | --- | --- | --- | --- | --- | --- | --- | --- | --- | --- | --- | --- | --- | --- | --- | --- | --- | --- | --- | --- | --- | --- | --- | --- | --- | --- | --- | --- | --- | --- | --- | --- | --- | --- | --- | --- | --- | --- | --- | --- | --- | --- | --- | --- | --- | --- | --- | --- | --- | --- | --- | --- | --- | --- | --- | --- | --- | --- | --- | --- | --- | --- | --- | --- | --- | --- | --- | --- | --- | --- | --- | --- | --- | --- | --- | --- | --- | --- | --- | --- | --- | --- | --- | --- | --- | --- | --- | --- | --- | --- | --- | --- | --- | --- | --- | --- | --- | --- | --- | --- | --- | --- | --- | --- | --- | --- | --- | --- | --- | --- | --- | --- | --- | --- | --- | --- | --- | --- | --- | --- | --- | --- | --- | --- | --- | --- | --- | --- | --- | --- | --- | --- | --- | --- | --- | --- | --- | --- | --- | --- | --- | --- | --- | --- | --- | --- | --- | --- | --- | --- | --- | --- | --- | --- | --- | --- | --- | --- | --- | --- | --- | --- | --- | --- | --- | --- | --- | --- | --- | --- | --- | --- | --- | --- | --- | --- | --- | --- | --- | --- | --- | --- | --- | --- | --- | --- | --- | --- | --- | --- | --- | --- | --- | --- | --- | --- | --- | --- | --- | --- | --- | --- | --- | --- | --- | --- | --- | --- | --- | --- | --- | --- | --- | --- | --- | --- | --- | --- | --- | --- | --- | --- | --- | --- | --- | --- | --- | --- | --- | --- | --- | --- | --- | --- | --- | --- | --- | --- | --- | --- | --- | --- | --- | --- | --- | --- | --- | --- | --- | --- | --- | --- | --- | --- | --- | --- | --- | --- | --- | --- | --- | --- | --- | --- | --- | --- | --- | --- | --- | --- | --- | --- | --- | --- | --- | --- | --- | --- | --- | --- | --- | --- | --- | --- | --- | --- | --- | --- | --- | --- | --- | --- | --- | --- | --- | --- | --- | --- | --- | --- | --- | --- | --- | --- | --- | --- | --- | --- | --- | --- | --- | --- | --- | --- | --- | --- | --- | --- | --- | --- | --- | --- | --- | --- | --- | --- | --- | --- | --- | --- | --- | --- | --- | --- | --- | --- | --- | --- | --- | --- | --- | --- | --- | --- | --- | --- | --- | --- | --- | --- | --- | --- | --- | --- | --- | --- | --- | --- | --- | --- | --- | --- | --- | --- | --- | --- | --- | --- | --- | --- | --- | --- | --- | --- | --- | --- | --- | --- | --- | --- | --- | --- | --- | --- | --- | --- | --- | --- | --- | --- | --- | --- | --- | --- | --- | --- | --- | --- | --- | --- | --- | --- | --- | --- | --- | --- | --- | --- | --- | --- | --- | --- | --- | --- | --- | --- | --- | --- | --- | --- | --- | --- | --- | --- | --- | --- | --- | --- | --- | --- | --- | --- | --- | --- | --- | --- | --- | --- | --- | --- | --- | --- | --- | --- | --- | --- | --- | --- | --- | --- | --- | --- | --- | --- | --- | --- | --- | --- | --- | --- | --- | --- | --- | --- | --- | --- | --- | --- | --- | --- | --- | --- | --- | --- | --- | --- | --- | --- | --- | --- | --- | --- | --- | --- | --- | --- | --- | --- | --- | --- | --- | --- | --- | --- | --- | --- | --- | --- | --- | --- | --- | --- | --- | --- | --- | --- | --- | --- | --- | --- | --- | --- | --- | --- | --- | --- | --- | --- | --- | --- | --- | --- | --- | --- | --- | --- | --- | --- | --- | --- | --- | --- | --- | --- | --- | --- | --- | --- | --- | --- | --- | --- | --- | --- | --- | --- | --- | --- | --- | --- | --- | --- | --- | --- | --- | --- | --- | --- | --- | --- | --- | --- | --- | --- | --- | --- | --- | --- | --- | --- | --- | --- | --- | --- | --- | --- | --- | --- | --- | --- | --- | --- | --- | --- | --- | --- | --- | --- | --- | --- | --- | --- | --- | --- | --- | --- | --- | --- | --- | --- | --- | --- | --- | --- | --- | --- | --- | --- | --- | --- | --- | --- | --- | --- | --- | --- | --- | --- | --- | --- | --- | --- | --- | --- | --- | --- | --- | --- | --- | --- | --- | --- | --- | --- | --- | --- | --- | --- | --- | --- | --- | --- | --- | --- | --- | --- | --- | --- | --- | --- | --- | --- | --- | --- | --- | --- | --- | --- | --- | --- | --- | --- | --- | --- | --- | --- | --- | --- | --- | --- | --- | --- | --- | --- | --- | --- | --- | --- | --- | --- | --- | --- | --- | --- | --- | --- | --- | --- | --- | --- | --- | --- | --- | --- | --- | --- | --- | --- | --- | --- | --- | --- | --- | --- | --- | --- | --- | --- | --- | --- | --- | --- | --- | --- | --- | --- | --- | --- | --- | --- | --- | --- | --- | --- | --- | --- | --- | --- | --- | --- | --- | --- | --- | --- | --- | --- | --- | --- | --- | --- | --- | --- | --- | --- | --- | --- | --- | --- | --- | --- | --- | --- | --- | --- | --- | --- | --- | --- | --- | --- | --- | --- | --- | --- | --- | --- | --- | --- | --- | --- | --- | --- | --- | --- | --- | --- | --- | --- | --- | --- | --- | --- | --- | --- | --- | --- | --- | --- | --- | --- | --- | --- | --- | --- | --- | --- | --- | --- | --- | --- | --- | --- | --- | --- | --- | --- | --- | --- | --- | --- | --- | --- | --- | --- | --- | --- | --- | --- | --- | --- | --- | --- | --- | --- | --- | --- | --- | --- | --- | --- | --- | --- | --- | --- | --- | --- | --- | --- | --- | --- | --- | --- | --- | --- | --- | --- | --- | --- | --- | --- | --- | --- | --- | --- | --- | --- | --- | --- | --- | --- | --- | --- | --- | --- | --- | --- | --- | --- | --- | --- | --- | --- | --- | --- | --- | --- | --- | --- | --- | --- | --- | --- | --- | --- | --- | --- | --- | --- | --- | --- | --- | --- | --- | --- | --- | --- | --- | --- | --- | --- | --- | --- | --- | --- | --- | --- | --- | --- | --- | --- | --- | --- | --- | --- | --- | --- | --- | --- | --- | --- | --- | --- | --- | --- | --- | --- | --- | --- | --- | --- | --- | --- | --- | --- | --- | --- | --- | --- | --- | --- | --- | --- | --- | --- | --- | --- | --- | --- | --- | --- | --- | --- | --- | --- | --- | --- | --- | --- | --- | --- | --- | --- | --- | --- | --- | --- | --- | --- | --- | --- | --- | --- | --- | --- | --- | --- | --- | --- | --- | --- | --- | --- | --- | --- | --- | --- | --- | --- | --- | --- | --- | --- | --- | --- | --- | --- | --- | --- | --- | --- | --- | --- | --- | --- | --- | --- | --- | --- | --- | --- | --- | --- | --- | --- | --- | --- | --- | --- | --- | --- | --- | --- | --- | --- | --- | --- | --- | --- | --- | --- | --- | --- | --- | --- | --- | --- | --- | --- | --- | --- | --- | --- | --- | --- | --- | --- | --- | --- | --- | --- | --- | --- | --- | --- | --- | --- | --- | --- | --- | --- | --- | --- | --- | --- | --- | --- | --- | --- | --- | --- | --- | --- | --- | --- | --- | --- | --- | --- | --- | --- | --- | --- | --- | --- | --- | --- | --- | --- | --- | --- | --- | --- | --- | --- | --- | --- | --- | --- | --- | --- | --- | --- | --- | --- | --- | --- | --- | --- | --- | --- | --- | --- | --- | --- | --- | --- | --- | --- | --- | --- | --- | --- | --- | --- | --- | --- | --- | --- | --- | --- | --- | --- | --- | --- | --- | --- | --- | --- | --- | --- | --- | --- | --- | --- | --- | --- | --- | --- | --- | --- | --- | --- | --- | --- | --- | --- | --- | --- | --- | --- | --- | --- | --- | --- | --- | --- | --- | --- | --- | --- | --- | --- | --- | --- | --- | --- | --- | --- | --- | --- | --- | --- | --- | --- | --- | --- | --- | --- | --- | --- | --- | --- | --- | --- | --- | --- | --- | --- | --- | --- | --- | --- | --- | --- | --- | --- | --- | --- | --- | --- | --- | --- | --- | --- | --- | --- | --- | --- | --- | --- | --- | --- | --- | --- | --- | --- | --- | --- | --- | --- | --- | --- | --- | --- | --- | --- | --- | --- | --- | --- | --- | --- | --- | --- | --- | --- | --- | --- | --- | --- | --- | --- | --- | --- | --- | --- | --- | --- | --- | --- | --- | --- | --- | --- | --- | --- | --- | --- | --- | --- | --- | --- | --- | --- | --- | --- | --- | --- | --- | --- | --- | --- | --- | --- | --- | --- | --- | --- | --- | --- | --- | --- | --- | --- | --- | --- | --- | --- | --- | --- | --- | --- | --- | --- | --- | --- | --- | --- | --- | --- | --- | --- | --- | --- | --- | --- | --- | --- | --- | --- | --- | --- | --- | --- | --- | --- | --- | --- | --- | --- | --- | --- | --- | --- | --- | --- | --- | --- | --- | --- | --- | --- | --- | --- | --- | --- | --- | --- | --- | --- | --- | --- | --- | --- | --- | --- | --- | --- | --- | --- | --- | --- | --- | --- | --- | --- | --- | --- | --- | --- | --- | --- | --- | --- | --- | --- | --- | --- | --- | --- | --- | --- | --- | --- | --- | --- | --- | --- | --- | --- | --- | --- | --- | --- | --- | --- | --- | --- | --- | --- | --- | --- | --- | --- | --- | --- | --- | --- | --- | --- | --- | --- | --- | --- | --- | --- | --- | --- | --- | --- | --- | --- | --- | --- | --- | --- | --- | --- | --- | --- | --- | --- | --- | --- | --- | --- | --- | --- | --- | --- | --- | --- | --- | --- | --- | --- | --- | --- | --- | --- | --- | --- | --- | --- | --- | --- | --- | --- | --- | --- | --- | --- | --- | --- | --- | --- | --- | --- | --- | --- | --- | --- | --- | --- | --- | --- | --- | --- | --- | --- | --- | --- | --- | --- | --- | --- | --- | --- | --- | --- | --- | --- | --- | --- | --- | --- | --- | --- | --- | --- | --- | --- | --- | --- | --- | --- | --- | --- | --- | --- | --- | --- | --- | --- | --- | --- | --- | --- | --- | --- | --- | --- | --- | --- | --- | --- | --- | --- | --- | --- | --- | --- | --- | --- | --- | --- | --- | --- | --- | --- | --- | --- | --- | --- | --- | --- | --- | --- | --- | --- | --- | --- | --- | --- | --- | --- | --- | --- | --- | --- | --- | --- | --- | --- | --- | --- | --- | --- | --- | --- | --- | --- | --- | --- | --- | --- | --- | --- | --- | --- | --- | --- | --- | --- | --- | --- | --- | --- | --- | --- | --- | --- | --- | --- | --- | --- | --- | --- | --- | --- | --- | --- | --- | --- | --- | --- | --- | --- | --- | --- | --- | --- | --- | --- | --- | --- | --- | --- | --- | --- | --- | --- | --- | --- | --- | --- | --- | --- | --- | --- | --- | --- | --- | --- | --- | --- | --- | --- | --- | --- | --- | --- | --- | --- | --- | --- | --- | --- | --- | --- | --- | --- | --- | --- | --- | --- | --- | --- | --- | --- | --- | --- | --- | --- | --- | --- | --- | --- | --- | --- | --- | --- | --- | --- | --- | --- | --- | --- | --- | --- | --- | --- | --- | --- | --- | --- | --- | --- | --- | --- | --- | --- | --- | --- | --- | --- | --- | --- | --- | --- | --- | --- | --- | --- | --- | --- | --- | --- | --- | --- | --- | --- | --- | --- | --- | --- | --- | --- | --- | --- | --- | --- | --- | --- | --- | --- | --- | --- | --- | --- | --- | --- | --- | --- | --- | --- | --- | --- | --- | --- | --- | --- | --- | --- | --- | --- | --- | --- | --- | --- | --- | --- | --- | --- | --- | --- | --- | --- | --- | --- | --- | --- | --- | --- | --- | --- | --- | --- | --- | --- | --- | --- | --- | --- | --- | --- | --- | --- | --- | --- | --- | --- | --- | --- | --- | --- | --- | --- | --- | --- | --- | --- | --- | --- | --- | --- | --- | --- | --- | --- | --- | --- | --- | --- | --- | --- | --- | --- | --- | --- | --- | --- | --- | --- | --- | --- | --- | --- | --- | --- | --- | --- | --- | --- | --- | --- | --- | --- | --- | --- | --- | --- | --- | --- | --- | --- | --- | --- | --- | --- | --- | --- | --- | --- | --- | --- | --- | --- | --- | --- | --- | --- | --- | --- | --- | --- | --- | --- | --- | --- | --- | --- | --- | --- | --- | --- | --- | --- | --- | --- | --- | --- | --- | --- | --- | --- | --- | --- | --- | --- | --- | --- | --- | --- | --- | --- | --- | --- | --- | --- | --- | --- | --- | --- | --- | --- | --- | --- | --- | --- | --- | --- | --- | --- | --- | --- | --- | --- | --- | --- | --- | --- | --- | --- | --- | --- | --- | --- | --- | --- | --- | --- | --- | --- | --- | --- | --- | --- | --- | --- | --- | --- | --- | --- | --- | --- | --- | --- | --- | --- | --- | --- | --- | --- | --- | --- | --- | --- | --- | --- | --- | --- | --- | --- | --- | --- | --- | --- | --- | --- | --- | --- | --- | --- | --- | --- | --- | --- | --- | --- | --- | --- | --- | --- | --- | --- | --- | --- | --- | --- | --- | --- | --- | --- | --- | --- | --- | --- | --- | --- | --- | --- | --- | --- | --- | --- | --- | --- | --- | --- | --- | --- | --- | --- | --- | --- | --- | --- | --- | --- | --- | --- | --- | --- | --- | --- | --- | --- | --- | --- | --- | --- | --- | --- | --- | --- | --- | --- | --- | --- | --- | --- | --- | --- | --- | --- | --- | --- | --- | --- | --- | --- | --- | --- | --- | --- | --- | --- | --- | --- | --- | --- | --- | --- | --- | --- | --- | --- | --- | --- | --- | --- | --- | --- | --- | --- | --- | --- | --- | --- | --- | --- | --- | --- | --- | --- | --- | --- | --- | --- | --- | --- | --- | --- | --- | --- | --- | --- | --- | --- | --- | --- | --- | --- | --- | --- | --- | --- | --- | --- | --- | --- | --- | --- | --- | --- | --- | --- | --- | --- | --- | --- | --- | --- | --- | --- | --- | --- | --- | --- | --- | --- | --- | --- | --- | --- | --- | --- | --- | --- | --- | --- | --- | --- | --- | --- | --- | --- | --- | --- | --- | --- | --- | --- | --- | --- | --- | --- | --- | --- | --- | --- | --- | --- | --- | --- | --- | --- | --- | --- | --- | --- | --- | --- | --- | --- | --- | --- | --- | --- | --- | --- | --- | --- | --- | --- | --- | --- | --- | --- | --- | --- | --- | --- | --- | --- | --- | --- | --- | --- | --- | --- | --- | --- | --- | --- | --- | --- | --- | --- | --- | --- | --- | --- | --- | --- | --- | --- | --- | --- | --- | --- | --- | --- | --- | --- | --- | --- | --- | --- | --- | --- | --- | --- | --- | --- | --- | --- | --- | --- | --- | --- | --- | --- | --- | --- | --- | --- | --- | --- | --- | --- | --- | --- | --- | --- | --- | --- | --- | --- | --- | --- | --- | --- | --- | --- | --- | --- | --- | --- | --- | --- | --- | --- | --- | --- | --- | --- | --- | --- | --- | --- | --- | --- | --- | --- | --- | --- | --- | --- | --- | --- | --- | --- | --- | --- | --- | --- | --- | --- | --- | --- | --- | --- | --- | --- | --- | --- | --- | --- | --- | --- | --- | --- | --- | --- | --- | --- | --- | --- | --- | --- | --- | --- | --- | --- | --- | --- | --- | --- | --- | --- | --- | --- | --- | --- | --- | --- | --- | --- | --- | --- | --- | --- | --- | --- | --- | --- | --- | --- | --- | --- | --- | --- | --- | --- | --- | --- | --- | --- | --- | --- | --- | --- | --- | --- | --- | --- | --- | --- | --- | --- | --- | --- | --- | --- | --- | --- | --- | --- | --- | --- | --- | --- | --- | --- | --- | --- | --- | --- | --- | --- | --- | --- | --- | --- | --- | --- | --- | --- | --- | --- | --- | --- | --- | --- | --- | --- | --- | --- | --- | --- | --- | --- | --- | --- | --- | --- | --- | --- | --- | --- | --- | --- | --- | --- | --- | --- | --- | --- | --- | --- | --- | --- | --- | --- | --- | --- | --- | --- | --- | --- | --- | --- | --- | --- | --- | --- | --- | --- | --- | --- | --- | --- | --- | --- | --- | --- | --- | --- | --- | --- | --- | --- | --- | --- | --- | --- | --- | --- | --- | --- | --- | --- | --- | --- | --- | --- | --- | --- | --- | --- | --- | --- | --- | --- | --- | --- | --- | --- | --- | --- | --- | --- | --- | --- | --- | --- | --- | --- | --- | --- | --- | --- | --- | --- | --- | --- | --- | --- | --- | --- | --- | --- | --- | --- | --- | --- | --- | --- | --- | --- | --- | --- | --- | --- | --- | --- | --- | --- | --- | --- | --- | --- | --- | --- | --- | --- | --- | --- | --- | --- | --- | --- | --- | --- | --- | --- | --- | --- | --- | --- | --- | --- | --- | --- | --- | --- | --- | --- | --- | --- | --- | --- | --- | --- | --- | --- | --- | --- | --- | --- | --- | --- | --- | --- | --- | --- | --- | --- | --- | --- | --- | --- | --- | --- | --- | --- | --- | --- | --- | --- | --- | --- | --- | --- | --- | --- | --- | --- | --- | --- | --- | --- | --- | --- | --- | --- | --- | --- | --- | --- | --- | --- | --- | --- | --- | --- | --- | --- | --- | --- | --- | --- | --- | --- | --- | --- | --- | --- | --- | --- | --- | --- | --- | --- | --- | --- | --- | --- | --- | --- | --- | --- | --- | --- | --- | --- | --- | --- | --- | --- | --- | --- | --- | --- | --- | --- | --- | --- | --- | --- | --- | --- | --- | --- | --- | --- | --- | --- | --- | --- | --- | --- | --- | --- | --- | --- | --- | --- | --- | --- | --- | --- | --- | --- | --- | --- | --- | --- | --- | --- | --- | --- | --- | --- | --- | --- | --- | --- | --- | --- | --- | --- | --- | --- | --- | --- | --- | --- | --- | --- | --- | --- | --- | --- | --- | --- | --- | --- | --- | --- | --- | --- | --- | --- | --- | --- | --- | --- | --- | --- | --- | --- | --- | --- | --- | --- | --- | --- | --- | --- | --- | --- | --- | --- | --- | --- | --- | --- | --- | --- | --- | --- | --- | --- | --- | --- | --- | --- | --- | --- | --- | --- | --- | --- | --- | --- | --- | --- | --- | --- | --- | --- | --- | --- | --- | --- | --- | --- | --- | --- | --- | --- | --- | --- | --- | --- | --- | --- | --- | --- | --- | --- | --- | --- | --- | --- | --- | --- | --- | --- | --- | --- | --- | --- | --- | --- | --- | --- | --- | --- | --- | --- | --- | --- | --- | --- | --- | --- | --- | --- | --- | --- | --- | --- | --- | --- | --- | --- | --- | --- | --- | --- | --- | --- | --- | --- | --- | --- | --- | --- | --- | --- | --- | --- | --- | --- | --- | --- | --- | --- | --- | --- | --- | --- | --- | --- | --- | --- | --- | --- | --- | --- | --- | --- | --- | --- | --- | --- | --- | --- | --- | --- | --- | --- | --- | --- | --- | --- | --- | --- | --- | --- | --- | --- | --- | --- | --- | --- | --- | --- | --- | --- | --- | --- | --- | --- | --- | --- | --- | --- | --- | --- | --- | --- | --- | --- | --- | --- | --- | --- | --- | --- | --- | --- | --- | --- | --- | --- | --- | --- | --- | --- | --- | --- | --- | --- | --- | --- | --- | --- | --- | --- | --- | --- | --- | --- | --- | --- | --- | --- | --- | --- | --- | --- | --- | --- | --- | --- | --- | --- | --- | --- | --- | --- | --- | --- | --- | --- | --- | --- | --- | --- | --- | --- | --- | --- | --- | --- | --- | --- | --- | --- | --- | --- | --- | --- | --- | --- | --- | --- | --- | --- | --- | --- | --- | --- | --- | --- | --- | --- | --- | --- | --- | --- | --- | --- | --- | --- | --- | --- | --- | --- | --- | --- | --- | --- | --- | --- | --- | --- | --- | --- | --- | --- | --- | --- | --- | --- | --- | --- | --- | --- | --- | --- | --- | --- | --- | --- | --- | --- | --- | --- | --- | --- | --- | --- | --- | --- | --- | --- | --- | --- | --- | --- | --- | --- | --- | --- | --- | --- | --- | --- | --- | --- | --- | --- | --- | --- | --- | --- | --- | --- | --- | --- | --- | --- | --- | --- | --- | --- | --- | --- | --- | --- | --- | --- | --- | --- | --- | --- | --- | --- | --- | --- | --- | --- | --- | --- | --- | --- | --- | --- | --- | --- | --- | --- | --- | --- | --- | --- | --- | --- | --- | --- | --- | --- | --- | --- | --- | --- | --- | --- | --- | --- | --- | --- | --- | --- | --- | --- | --- | --- | --- | --- | --- | --- | --- | --- | --- | --- | --- | --- | --- | --- | --- | --- | --- | --- | --- | --- | --- | --- | --- | --- | --- | --- | --- | --- | --- | --- | --- | --- | --- | --- | --- | --- | --- | --- | --- | --- | --- | --- | --- | --- | --- | --- | --- | --- | --- | --- | --- | --- | --- | --- | --- | --- | --- | --- | --- | --- | --- | --- | --- | --- | --- | --- | --- | --- | --- | --- | --- | --- | --- | --- | --- | --- | --- | --- | --- | --- | --- | --- | --- | --- | --- | --- | --- | --- | --- | --- | --- | --- | --- | --- | --- | --- | --- | --- | --- | --- | --- | --- | --- | --- | --- | --- | --- | --- | --- | --- | --- | --- | --- | --- | --- | --- | --- | --- | --- | --- | --- | --- | --- | --- | --- | --- | --- | --- | --- | --- | --- | --- | --- | --- | --- | --- | --- | --- | --- | --- | --- | --- | --- | --- | --- | --- | --- | --- | --- | --- | --- | --- | --- | --- | --- | --- | --- | --- | --- | --- | --- | --- | --- | --- | --- | --- | --- | --- | --- | --- | --- | --- | --- | --- | --- | --- | --- | --- | --- | --- | --- | --- | --- | --- | --- | --- | --- | --- | --- | --- | --- | --- | --- | --- | --- | --- | --- | --- | --- | --- | --- | --- | --- | --- | --- | --- | --- | --- | --- | --- | --- | --- | --- | --- | --- | --- | --- | --- | --- | --- | --- | --- | --- | --- | --- | --- | --- | --- | --- | --- | --- | --- | --- | --- | --- | --- | --- | --- | --- | --- | --- | --- | --- | --- | --- | --- | --- | --- | --- | --- | --- | --- | --- | --- | --- | --- | --- | --- | --- | --- | --- | --- | --- | --- | --- | --- | --- | --- | --- | --- | --- | --- | --- | --- | --- | --- | --- | --- | --- | --- | --- | --- | --- | --- | --- | --- | --- | --- | --- | --- | --- | --- | --- | --- | --- | --- | --- | --- | --- | --- | --- | --- | --- | --- | --- | --- | --- | --- | --- | --- | --- | --- | --- | --- | --- | --- | --- | --- | --- | --- | --- | --- | --- | --- | --- | --- | --- | --- | --- | --- | --- | --- | --- | --- | --- | --- | --- | --- | --- | --- | --- | --- | --- | --- | --- | --- | --- | --- | --- | --- | --- | --- | --- | --- | --- | --- | --- | --- | --- | --- | --- | --- | --- | --- | --- | --- | --- | --- | --- | --- | --- | --- | --- | --- | --- | --- | --- | --- | --- | --- | --- | --- | --- | --- | --- | --- | --- | --- | --- | --- | --- | --- | --- | --- | --- | --- | --- | --- | --- | --- | --- | --- | --- | --- | --- | --- | --- | --- | --- | --- | --- | --- | --- | --- | --- | --- | --- | --- | --- | --- | --- | --- | --- | --- | --- | --- | --- | --- | --- | --- | --- | --- | --- | --- | --- | --- | --- | --- | --- | --- | --- | --- | --- | --- | --- | --- | --- | --- | --- | --- | --- | --- | --- | --- | --- | --- | --- | --- | --- | --- | --- | --- | --- | --- | --- | --- | --- | --- | --- | --- | --- | --- | --- | --- | --- | --- | --- | --- | --- | --- | --- | --- | --- | --- | --- | --- | --- | --- | --- | --- | --- | --- | --- | --- | --- | --- | --- | --- | --- | --- | --- | --- | --- | --- | --- | --- | --- | --- | --- | --- | --- | --- | --- | --- | --- | --- | --- | --- | --- | --- | --- | --- | --- | --- | --- | --- | --- | --- | --- | --- | --- | --- | --- | --- | --- | --- | --- | --- | --- | --- | --- | --- | --- | --- | --- | --- | --- | --- | --- | --- | --- | --- | --- | --- | --- | --- | --- | --- | --- | --- | --- | --- | --- | --- | --- | --- | --- | --- | --- | --- | --- | --- | --- | --- | --- | --- | --- | --- | --- | --- | --- | --- | --- | --- | --- | --- | --- | --- | --- | --- | --- | --- | --- | --- | --- | --- | --- | --- | --- | --- | --- | --- | --- | --- | --- | --- | --- | --- | --- | --- | --- | --- | --- | --- | --- | --- | --- | --- | --- | --- | --- | --- | --- | --- | --- | --- | --- | --- | --- | --- | --- | --- | --- | --- | --- | --- | --- | --- | --- | --- | --- | --- | --- | --- | --- | --- | --- | --- | --- | --- | --- | --- | --- | --- | --- | --- | --- | --- | --- | --- | --- | --- | --- | --- | --- | --- | --- | --- | --- | --- | --- | --- | --- | --- | --- | --- | --- | --- | --- | --- | --- | --- | --- | --- | --- | --- | --- | --- | --- | --- | --- | --- | --- | --- | --- | --- | --- | --- | --- | --- | --- | --- | --- | --- | --- | --- | --- | --- | --- | --- | --- | --- | --- | --- | --- | --- | --- | --- | --- | --- | --- | --- | --- | --- | --- | --- | --- | --- | --- | --- | --- | --- | --- | --- | --- | --- | --- | --- | --- | --- | --- | --- | --- | --- | --- | --- | --- | --- | --- | --- | --- | --- | --- | --- | --- | --- | --- | --- | --- | --- | --- | --- | --- | --- | --- | --- | --- | --- | --- | --- | --- | --- | --- | --- | --- | --- | --- | --- | --- | --- | --- | --- | --- | --- | --- | --- | --- | --- | --- | --- | --- | --- | --- | --- | --- | --- | --- | --- | --- | --- | --- | --- | --- | --- | --- | --- | --- | --- | --- | --- | --- | --- | --- | --- | --- | --- | --- | --- | --- | --- | --- | --- | --- | --- | --- | --- | --- | --- | --- | --- | --- | --- | --- | --- | --- | --- | --- | --- | --- | --- | --- | --- | --- | --- | --- | --- | --- | --- | --- | --- | --- | --- | --- | --- | --- | --- | --- | --- | --- | --- | --- | --- | --- | --- | --- | --- | --- | --- | --- | --- | --- | --- | --- | --- | --- | --- | --- | --- | --- | --- | --- | --- | --- | --- | --- | --- | --- | --- | --- | --- | --- | --- | --- | --- | --- | --- | --- | --- | --- | --- | --- | --- | --- | --- | --- | --- | --- | --- | --- | --- | --- | --- | --- | --- | --- | --- | --- | --- | --- | --- | --- | --- | --- | --- | --- | --- | --- | --- | --- | --- | --- | --- | --- | --- | --- | --- | --- | --- | --- | --- | --- | --- | --- | --- | --- | --- | --- | --- | --- | --- | --- | --- | --- | --- | --- | --- | --- | --- | --- | --- | --- | --- | --- | --- | --- | --- | --- | --- | --- | --- | --- | --- | --- | --- | --- | --- | --- | --- | --- | --- | --- | --- | --- | --- | --- | --- | --- | --- | --- | --- | --- | --- | --- | --- | --- | --- | --- | --- | --- | --- | --- | --- | --- | --- | --- | --- | --- | --- | --- | --- | --- | --- | --- | --- | --- | --- | --- | --- | --- | --- | --- | --- | --- | --- | --- | --- | --- | --- | --- | --- | --- | --- | --- | --- | --- | --- | --- | --- | --- | --- | --- | --- | --- | --- | --- | --- | --- | --- | --- | --- | --- | --- | --- | --- | --- | --- | --- | --- | --- | --- | --- | --- | --- | --- | --- | --- | --- | --- | --- | --- | --- | --- | --- | --- | --- | --- | --- | --- | --- | --- | --- | --- | --- | --- | --- | --- | --- | --- | --- | --- | --- | --- | --- | --- | --- | --- | --- | --- | --- | --- | --- | --- | --- | --- | --- | --- | --- | --- | --- | --- | --- | --- | --- | --- | --- | --- | --- | --- | --- | --- | --- | --- | --- | --- | --- | --- | --- | --- | --- | --- | --- | --- | --- | --- | --- | --- | --- | --- | --- | --- | --- | --- | --- | --- | --- | --- | --- | --- | --- | --- | --- | --- | --- | --- | --- | --- | --- | --- | --- | --- | --- | --- | --- | --- | --- | --- | --- | --- | --- | --- | --- | --- | --- | --- | --- | --- | --- | --- | --- | --- | --- | --- | --- | --- | --- | --- | --- | --- | --- | --- | --- | --- | --- | --- | --- | --- | --- | --- | --- | --- | --- | --- | --- | --- | --- | --- | --- | --- | --- | --- | --- | --- | --- | --- | --- | --- | --- | --- | --- | --- | --- | --- | --- | --- | --- | --- | --- | --- | --- | --- | --- | --- | --- | --- | --- | --- | --- | --- | --- | --- | --- | --- | --- | --- | --- | --- | --- | --- | --- | --- | --- | --- | --- | --- | --- | --- | --- | --- | --- | --- | --- | --- | --- | --- | --- | --- | --- | --- | --- | --- | --- | --- | --- | --- | --- | --- | --- | --- | --- | --- | --- | --- | --- | --- | --- | --- | --- | --- | --- | --- | --- | --- | --- | --- | --- | --- | --- | --- | --- | --- | --- | --- | --- | --- | --- | --- | --- | --- | --- | --- | --- | --- | --- | --- | --- | --- | --- | --- | --- | --- | --- | --- | --- | --- | --- | --- | --- | --- | --- | --- | --- | --- | --- | --- | --- | --- | --- | --- | --- | --- | --- | --- | --- | --- | --- | --- | --- | --- | --- | --- | --- | --- | --- | --- | --- | --- | --- | --- | --- | --- | --- | --- | --- | --- | --- | --- | --- | --- | --- | --- | --- | --- | --- | --- | --- | --- | --- | --- | --- | --- | --- | --- | --- | --- | --- | --- | --- | --- | --- | --- | --- | --- | --- | --- | --- | --- | --- | --- | --- | --- | --- | --- | --- | --- | --- | --- | --- | --- | --- | --- | --- | --- | --- | --- | --- | --- | --- | --- | --- | --- | --- | --- | --- | --- | --- | --- | --- | --- | --- | --- | --- | --- | --- | --- | --- | --- | --- | --- | --- | --- | --- | --- | --- | --- | --- | --- | --- | --- | --- | --- | --- | --- | --- | --- | --- | --- | --- | --- | --- | --- | --- | --- | --- | --- | --- | --- | --- | --- | --- | --- | --- | --- | --- | --- | --- | --- | --- | --- | --- | --- | --- | --- | --- | --- | --- | --- | --- | --- | --- | --- | --- | --- | --- | --- | --- | --- | --- | --- | --- | --- | --- | --- | --- | --- | --- | --- | --- | --- | --- | --- | --- | --- | --- | --- | --- | --- | --- | --- | --- | --- | --- | --- | --- | --- | --- | --- | --- | --- | --- | --- | --- | --- | --- | --- | --- | --- | --- | --- | --- | --- | --- | --- | --- | --- | --- | --- | --- | --- | --- | --- | --- | --- | --- | --- | --- | --- | --- | --- | --- | --- | --- | --- | --- | --- | --- | --- | --- | --- | --- | --- | --- | --- | --- | --- | --- | --- | --- | --- | --- | --- | --- | --- | --- | --- | --- | --- | --- | --- | --- | --- | --- | --- | --- | --- | --- | --- | --- | --- | --- | --- | --- | --- | --- | --- | --- | --- | --- | --- | --- | --- | --- | --- | --- | --- | --- | --- | --- | --- | --- | --- | --- | --- | --- | --- | --- | --- | --- | --- | --- | --- | --- | --- | --- | --- | --- | --- | --- | --- | --- | --- | --- | --- | --- | --- | --- | --- | --- | --- | --- | --- | --- | --- | --- | --- | --- | --- | --- | --- | --- | --- | --- | --- | --- | --- | --- | --- | --- | --- | --- | --- | --- | --- | --- | --- | --- | --- | --- | --- | --- | --- | --- | --- | --- | --- | --- | --- | --- | --- | --- | --- | --- | --- | --- | --- | --- | --- | --- | --- | --- | --- | --- | --- | --- | --- | --- | --- | --- | --- | --- | --- | --- | --- | --- | --- | --- | --- | --- | --- | --- | --- | --- | --- | --- | --- | --- | --- | --- | --- | --- | --- | --- | --- | --- | --- | --- | --- | --- | --- | --- | --- | --- | --- | --- | --- | --- | --- | --- | --- | --- | --- | --- | --- | --- | --- | --- | --- | --- | --- | --- | --- | --- | --- | --- | --- | --- | --- | --- | --- | --- | --- | --- | --- | --- | --- | --- | --- | --- | --- | --- | --- | --- | --- | --- | --- | --- | --- | --- | --- | --- | --- | --- | --- | --- | --- | --- | --- | --- | --- | --- | --- | --- | --- | --- | --- | --- | --- | --- | --- | --- | --- | --- | --- | --- | --- | --- | --- | --- | --- | --- | --- | --- | --- | --- | --- | --- | --- | --- | --- | --- | --- | --- | --- | --- | --- | --- | --- | --- | --- | --- | --- | --- | --- | --- | --- | --- | --- | --- | --- | --- | --- | --- | --- | --- | --- | --- | --- | --- | --- | --- | --- | --- | --- | --- | --- | --- | --- | --- | --- | --- | --- | --- | --- | --- | --- | --- | --- | --- | --- | --- | --- | --- | --- | --- | --- | --- | --- | --- | --- | --- | --- | --- | --- | --- | --- | --- | --- | --- | --- | --- | --- | --- | --- | --- | --- | --- | --- | --- | --- | --- | --- | --- | --- | --- | --- | --- | --- | --- | --- | --- | --- | --- | --- | --- | --- | --- | --- | --- | --- | --- | --- | --- | --- | --- | --- | --- | --- | --- | --- | --- | --- | --- | --- | --- | --- | --- | --- | --- | --- | --- | --- | --- | --- | --- | --- | --- | --- | --- | --- | --- | --- | --- | --- | --- | --- | --- | --- | --- | --- | --- | --- | --- | --- | --- | --- | --- | --- | --- | --- | --- | --- | --- | --- | --- | --- | --- | --- | --- | --- | --- | --- | --- | --- | --- | --- | --- | --- | --- | --- | --- | --- | --- | --- | --- | --- | --- | --- | --- | --- | --- | --- | --- | --- | --- | --- | --- | --- | --- | --- | --- | --- | --- | --- | --- | --- | --- | --- | --- | --- | --- | --- | --- | --- | --- | --- | --- | --- | --- | --- | --- | --- | --- | --- | --- | --- | --- | --- | --- | --- | --- | --- | --- | --- | --- | --- | --- | --- | --- | --- | --- | --- | --- | --- | --- | --- | --- | --- | --- | --- | --- | --- | --- | --- | --- | --- | --- | --- | --- | --- | --- | --- | --- | --- | --- | --- | --- | --- | --- | --- | --- | --- | --- | --- | --- | --- | --- | --- | --- | --- | --- | --- | --- | --- | --- | --- | --- | --- | --- | --- | --- | --- | --- | --- | --- | --- | --- | --- | --- | --- | --- | --- | --- | --- | --- | --- | --- | --- | --- | --- | --- | --- | --- | --- | --- | --- | --- | --- | --- | --- | --- | --- | --- | --- | --- | --- | --- | --- | --- | --- | --- | --- | --- | --- | --- | --- | --- | --- | --- | --- | --- | --- | --- | --- | --- | --- | --- | --- | --- | --- | --- | --- | --- | --- | --- | --- | --- | --- | --- | --- | --- | --- | --- | --- | --- | --- | --- | --- | --- | --- | --- | --- | --- | --- | --- | --- | --- | --- | --- | --- | --- | --- | --- | --- | --- | --- | --- | --- | --- | --- | --- | --- | --- | --- | --- | --- | --- | --- | --- | --- | --- | --- | --- | --- | --- | --- | --- | --- | --- | --- | --- | --- | --- | --- | --- | --- | --- | --- | --- | --- | --- | --- | --- | --- | --- | --- | --- | --- | --- | --- | --- | --- | --- | --- | --- | --- | --- | --- | --- | --- | --- | --- | --- | --- | --- | --- | --- | --- | --- | --- | --- | --- | --- | --- | --- | --- | --- | --- | --- | --- | --- | --- | --- | --- | --- | --- | --- | --- | --- | --- | --- | --- | --- | --- | --- | --- | --- | --- | --- | --- | --- | --- | --- | --- | --- | --- | --- | --- | --- | --- | --- | --- | --- | --- | --- | --- | --- | --- | --- | --- | --- | --- | --- | --- | --- | --- | --- | --- | --- | --- | --- | --- | --- | --- | --- | --- | --- | --- | --- | --- | --- | --- | --- | --- | --- | --- | --- | --- | --- | --- | --- | --- | --- | --- | --- | --- | --- | --- | --- | --- | --- | --- | --- | --- | --- | --- | --- | --- | --- | --- | --- | --- | --- | --- | --- | --- | --- | --- | --- | --- | --- | --- | --- | --- | --- | --- | --- | --- | --- | --- | --- | --- | --- | --- | --- | --- | --- | --- | --- | --- | --- | --- | --- | --- | --- | --- | --- | --- | --- | --- | --- | --- | --- | --- | --- | --- | --- | --- | --- | --- | --- | --- | --- | --- | --- | --- | --- | --- | --- | --- | --- | --- | --- | --- | --- | --- | --- | --- | --- | --- | --- | --- | --- | --- | --- | --- | --- | --- | --- | --- | --- | --- | --- | --- | --- | --- | --- | --- | --- | --- | --- | --- | --- | --- | --- | --- | --- | --- | --- | --- | --- | --- | --- | --- | --- | --- | --- | --- | --- | --- | --- | --- | --- | --- | --- | --- | --- | --- | --- | --- | --- | --- | --- | --- | --- | --- | --- | --- | --- | --- | --- | --- | --- | --- | --- | --- | --- | --- | --- | --- | --- | --- | --- | --- | --- | --- | --- | --- | --- | --- | --- | --- | --- | --- | --- | --- | --- | --- | --- | --- | --- | --- | --- | --- | --- | --- | --- | --- | --- | --- | --- | --- | --- | --- | --- | --- | --- | --- | --- | --- | --- | --- | --- | --- | --- | --- | --- | --- | --- | --- | --- | --- | --- | --- | --- | --- | --- | --- | --- | --- | --- | --- | --- | --- | --- | --- | --- | --- | --- | --- | --- | --- | --- | --- | --- | --- | --- | --- | --- | --- | --- | --- | --- | --- | --- | --- | --- | --- | --- | --- | --- | --- | --- | --- | --- | --- | --- | --- | --- | --- | --- | --- | --- | --- | --- | --- | --- | --- | --- | --- | --- | --- | --- | --- | --- | --- | --- | --- | --- | --- | --- | --- | --- | --- | --- | --- | --- | --- | --- | --- | --- | --- | --- | --- | --- | --- | --- | --- | --- | --- | --- | --- | --- | --- | --- | --- | --- | --- | --- | --- | --- | --- | --- | --- | --- | --- | --- | --- | --- | --- | --- | --- | --- | --- | --- | --- | --- | --- | --- | --- | --- | --- | --- | --- | --- | --- | --- | --- | --- | --- | --- | --- | --- | --- | --- | --- | --- | --- | --- | --- | --- | --- | --- | --- | --- | --- | --- | --- | --- | --- | --- | --- | --- | --- | --- | --- | --- | --- | --- | --- | --- | --- | --- | --- | --- | --- | --- | --- | --- | --- | --- | --- | --- | --- | --- | --- | --- | --- | --- | --- | --- | --- | --- | --- | --- | --- | --- | --- | --- | --- | --- | --- | --- | --- | --- | --- | --- | --- | --- | --- | --- | --- | --- | --- | --- | --- | --- | --- | --- | --- | --- | --- | --- | --- | --- | --- | --- | --- | --- | --- | --- | --- | --- | --- | --- | --- | --- | --- | --- | --- | --- | --- | --- | --- | --- | --- | --- | --- | --- | --- | --- | --- | --- | --- | --- | --- | --- | --- | --- | --- | --- | --- | --- | --- | --- | --- | --- | --- | --- | --- | --- | --- | --- | --- | --- | --- | --- | --- | --- | --- | --- | --- | --- | --- | --- | --- | --- | --- | --- | --- | --- | --- | --- | --- | --- | --- | --- | --- | --- | --- | --- | --- | --- | --- | --- | --- | --- | --- | --- | --- | --- | --- | --- | --- | --- | --- | --- | --- | --- | --- | --- | --- | --- | --- | --- | --- | --- | --- | --- | --- | --- | --- | --- | --- | --- | --- | --- | --- | --- | --- | --- | --- | --- | --- | --- | --- | --- | --- | --- | --- | --- | --- | --- | --- | --- | --- | --- | --- | --- | --- | --- | --- | --- | --- | --- | --- | --- | --- | --- | --- | --- | --- | --- | --- | --- | --- | --- | --- | --- | --- | --- | --- | --- | --- | --- | --- | --- | --- | --- | --- | --- | --- | --- | --- | --- | --- | --- | --- | --- | --- | --- | --- | --- | --- | --- | --- | --- | --- | --- | --- | --- | --- | --- | --- | --- | --- | --- | --- | --- | --- | --- | --- | --- | --- | --- | --- | --- | --- | --- | --- | --- | --- | --- | --- | --- | --- | --- | --- | --- | --- | --- | --- | --- | --- | --- | --- | --- | --- | --- | --- | --- | --- | --- | --- | --- | --- | --- | --- | --- | --- | --- | --- | --- | --- | --- | --- | --- | --- | --- | --- | --- | --- | --- | --- | --- | --- | --- | --- | --- | --- | --- | --- | --- | --- | --- | --- | --- | --- | --- | --- | --- | --- | --- | --- | --- | --- | --- | --- | --- | --- | --- | --- | --- | --- | --- | --- | --- | --- | --- | --- | --- | --- | --- | --- | --- | --- | --- | --- | --- | --- | --- | --- | --- | --- | --- | --- | --- | --- | --- | --- | --- | --- | --- | --- | --- | --- | --- | --- | --- | --- | --- | --- | --- | --- | --- | --- | --- | --- | --- | --- | --- | --- | --- | --- | --- | --- | --- | --- | --- | --- | --- | --- | --- | --- | --- | --- | --- | --- | --- | --- | --- | --- | --- | --- | --- | --- | --- | --- | --- | --- | --- | --- | --- | --- | --- | --- | --- | --- | --- | --- | --- | --- | --- | --- | --- | --- | --- | --- | --- | --- | --- | --- | --- | --- | --- | --- | --- | --- | --- | --- | --- | --- | --- | --- | --- | --- | --- | --- | --- | --- | --- | --- | --- | --- | --- | --- | --- | --- | --- | --- | --- | --- | --- | --- | --- | --- | --- | --- | --- | --- | --- | --- | --- | --- | --- | --- | --- | --- | --- | --- | --- | --- | --- | --- | --- | --- | --- | --- | --- | --- | --- | --- | --- | --- | --- | --- | --- | --- | --- | --- | --- | --- | --- | --- | --- | --- | --- | --- | --- | --- | --- | --- | --- | --- | --- | --- | --- | --- | --- | --- | --- | --- | --- | --- | --- | --- | --- | --- | --- | --- | --- | --- | --- | --- | --- | --- | --- | --- | --- | --- | --- | --- | --- | --- | --- | --- | --- | --- | --- | --- | --- | --- | --- | --- | --- | --- | --- | --- | --- | --- | --- | --- | --- | --- | --- | --- | --- | --- | --- | --- | --- | --- | --- | --- | --- | --- | --- | --- | --- | --- | --- | --- | --- | --- | --- | --- | --- | --- | --- | --- | --- | --- | --- | --- | --- | --- | --- | --- | --- | --- | --- | --- | --- | --- | --- | --- | --- | --- | --- | --- | --- | --- | --- | --- | --- | --- | --- | --- | --- | --- | --- | --- | --- | --- | --- | --- | --- | --- | --- | --- | --- | --- | --- | --- | --- | --- | --- | --- | --- | --- | --- | --- | --- | --- | --- | --- | --- | --- | --- | --- | --- | --- | --- | --- | --- | --- | --- | --- | --- | --- | --- | --- | --- | --- | --- | --- | --- | --- | --- | --- | --- | --- | --- | --- | --- | --- | --- | --- | --- | --- | --- | --- | --- | --- | --- | --- | --- | --- | --- | --- | --- | --- | --- | --- | --- | --- | --- | --- | --- | --- | --- | --- | --- | --- | --- | --- | --- | --- | --- | --- | --- | --- | --- | --- | --- | --- | --- | --- | --- | --- | --- | --- | --- | --- | --- | --- | --- | --- | --- | --- | --- | --- | --- | --- | --- | --- | --- | --- | --- | --- | --- | --- | --- | --- | --- | --- | --- | --- | --- | --- | --- | --- | --- | --- | --- | --- | --- | --- | --- | --- | --- | --- | --- | --- | --- | --- | --- | --- | --- | --- | --- | --- | --- | --- | --- | --- | --- | --- | --- | --- | --- | --- | --- | --- | --- | --- | --- | --- | --- | --- | --- | --- | --- | --- | --- | --- | --- | --- | --- | --- | --- | --- | --- | --- | --- | --- | --- | --- | --- | --- | --- | --- | --- | --- | --- | --- | --- | --- | --- | --- | --- | --- | --- | --- | --- | --- | --- | --- | --- | --- | --- | --- | --- | --- | --- | --- | --- | --- | --- | --- | --- | --- | --- | --- | --- | --- | --- | --- | --- | --- | --- | --- | --- | --- | --- | --- | --- | --- | --- | --- | --- | --- | --- | --- | --- | --- | --- | --- | --- | --- | --- | --- | --- | --- | --- | --- | --- | --- | --- | --- | --- | --- | --- | --- | --- | --- | --- | --- | --- | --- | --- | --- | --- | --- | --- | --- | --- | --- | --- | --- | --- | --- | --- | --- | --- | --- | --- | --- | --- | --- | --- | --- | --- | --- | --- | --- | --- | --- | --- | --- | --- | --- | --- | --- | --- | --- | --- | --- | --- | --- | --- | --- | --- | --- | --- | --- | --- | --- | --- | --- | --- | --- | --- | --- | --- | --- | --- | --- | --- | --- | --- | --- | --- | --- | --- | --- | --- | --- | --- | --- | --- | --- | --- | --- | --- | --- | --- | --- | --- | --- | --- | --- | --- | --- | --- | --- | --- | --- | --- | --- | --- | --- | --- | --- | --- | --- | --- | --- | --- | --- | --- | --- | --- | --- | --- | --- | --- | --- | --- | --- | --- | --- | --- | --- | --- | --- | --- | --- | --- | --- | --- | --- | --- | --- | --- | --- | --- | --- | --- | --- | --- | --- | --- | --- | --- | --- | --- | --- | --- | --- | --- | --- | --- | --- | --- | --- | --- | --- | --- | --- | --- | --- | --- | --- | --- | --- | --- | --- | --- | --- | --- | --- | --- | --- | --- | --- | --- | --- | --- | --- | --- | --- | --- | --- | --- | --- | --- | --- | --- | --- | --- | --- | --- | --- | --- | --- | --- | --- | --- | --- | --- | --- | --- | --- | --- | --- | --- | --- | --- | --- | --- | --- | --- | --- | --- | --- | --- | --- | --- | --- | --- | --- | --- | --- | --- | --- | --- | --- | --- | --- | --- | --- | --- | --- | --- | --- | --- | --- | --- | --- | --- | --- | --- | --- | --- | --- | --- | --- | --- | --- | --- | --- | --- | --- | --- | --- | --- | --- | --- | --- | --- | --- | --- | --- | --- | --- | --- | --- | --- | --- | --- | --- | --- | --- | --- | --- | --- | --- | --- | --- | --- | --- | --- | --- | --- | --- | --- | --- | --- | --- | --- | --- | --- |
|  |  |  |  |  |  |  |  |  |  |  |
|  | | | **Method A** |  | **Method B** | **OR** | **CI** | **p-val.** |  |  |
|  | | | | | | | | |  |  |
| Artifacts | | | DL-1 | vs. | DL-1-SD | 2.679 | [ 1.67; 4.31] | <0.001 |  |  |
|  |  |  | DL-1 | vs. | DL-2 | 0.640 | [ 0.56; 0.73] | <0.001 |  |  |
|  |  |  | DL-1 | vs. | AIDR | 0.849 | [ 0.70; 1.03] | 0.094 |  |  |
|  |  |  | DL-1-SD | vs. | DL-2 | 0.239 | [ 0.15; 0.39] | <0.001 |  |  |
|  |  |  | DL-1-SD | vs. | AIDR | 0.317 | [ 0.20; 0.50] | <0.001 |  |  |
|  |  |  | DL-2 | vs. | AIDR | 1.327 | [ 1.07; 1.65] | 0.011 |  |  |
|  | | | | | | | | |  |  |
| Nasopharynx | | | DL-1 | vs. | DL-1-SD | 0.388 | [ 0.25; 0.60] | <0.001 |  |  |
|  |  |  | DL-1 | vs. | DL-2 | 0.018 | [ 0.01; 0.03] | <0.001 |  |  |
|  |  |  | DL-1 | vs. | AIDR | 27.559 | [ 17.19; 44.18] | <0.001 |  |  |
|  |  |  | DL-1-SD | vs. | DL-2 | 0.046 | [ 0.03; 0.08] | <0.001 |  |  |
|  |  |  | DL-1-SD | vs. | AIDR | 71.070 | [ 40.21; 125.62] | <0.001 |  |  |
|  |  |  | DL-2 | vs. | AIDR | >999.999 | [ 816.46;>999.99] | <0.001 |  |  |
|  | | | | | | | | |  |  |
| Hypopharynx | | | DL-1 | vs. | DL-1-SD | 0.360 | [ 0.24; 0.54] | <0.001 |  |  |
|  |  |  | DL-1 | vs. | DL-2 | 0.019 | [ 0.01; 0.03] | <0.001 |  |  |
|  |  |  | DL-1 | vs. | AIDR | 25.090 | [ 16.10; 39.09] | <0.001 |  |  |
|  |  |  | DL-1-SD | vs. | DL-2 | 0.052 | [ 0.03; 0.08] | <0.001 |  |  |
|  | | | DL-1-SD | vs. | AIDR | 69.748 | [ 40.53; 120.03] | <0.001 |  |  |
|  | | | DL-2 | vs. | AIDR | >999.999 | [ 696.30;>999.99] | <0.001 |  |  |
|  | | |  |  |  |  |  |  |  |  |
| Oral cavity | | | DL-1 | vs. | DL-1-SD | 0.822 | [ 0.53; 1.26] | 0.372 |  |  |
|  | | | DL-1 | vs. | DL-2 | 0.364 | [ 0.30; 0.44] | <0.001 |  |  |
|  | | | DL-1 | vs. | AIDR | 2.185 | [ 1.83; 2.60] | <0.001 |  |  |
|  | | | DL-1-SD | vs. | DL-2 | 0.443 | [ 0.28; 0.70] | <0.001 |  |  |
|  | | | DL-1-SD | vs. | AIDR | 2.658 | [ 1.76; 4.02] | <0.001 |  |  |
|  | | | DL-2 | vs. | AIDR | 5.996 | [ 4.70; 7.65] | <0.001 |  |  |
|  | | |  |  |  |  |  |  |  |  |
| Salivary glands | | | DL-1 | vs. | DL-1-SD | 0.330 | [ 0.23; 0.48] | <0.001 |  |  |
|  | | | DL-1 | vs. | DL-2 | 0.037 | [ 0.02; 0.06] | <0.001 |  |  |
|  | | | DL-1 | vs. | AIDR | 18.102 | [ 11.75; 27.90] | <0.001 |  |  |
|  | | | DL-1-SD | vs. | DL-2 | 0.112 | [ 0.07; 0.17] | <0.001 |  |  |
|  | | | DL-1-SD | vs. | AIDR | 54.932 | [ 32.84; 91.88] | <0.001 |  |  |
|  | | | DL-2 | vs. | AIDR | 489.622 | [ 266.11; 900.86] | <0.001 |  |  |
| Lymphnodes | | | DL-1 | vs. | DL-1-SD | 0.331 | [ 0.22; 0.50] | <0.001 |  |  |
|  | | | DL-1 | vs. | DL-2 | 0.046 | [ 0.03; 0.07] | <0.001 |  |  |
|  | | | DL-1 | vs. | AIDR | 20.581 | [ 13.73; 30.86] | <0.001 |  |  |
|  | | | DL-1-SD | vs. | DL-2 | 0.139 | [ 0.09; 0.21] | <0.001 |  |  |
|  | | | DL-1-SD | vs. | AIDR | 62.090 | [ 36.95; 104.33] | <0.001 |  |  |
|  | | | DL-2 | vs. | AIDR | 447.749 | [ 258.69; 774.98] | <0.001 |  |  |
|  | | |  |  |  |  |  |  |  |  |
| pterygopalatine fossa | | | DL-1 | vs. | DL-1-SD | 0.325 | [ 0.21; 0.51] | <0.001 |  |  |
|  |  |  | DL-1 | vs. | DL-2 | 0.018 | [ 0.01; 0.03] | <0.001 |  |  |
|  |  |  | DL-1 | vs. | AIDR | 24.347 | [ 15.85; 37.40] | <0.001 |  |  |
|  | | | DL-1-SD | vs. | DL-2 | 0.056 | [ 0.04; 0.09] | <0.001 |  |  |
|  | | | DL-1-SD | vs. | AIDR | 74.895 | [ 42.68; 131.43] | <0.001 |  |  |
|  | | | DL-2 | vs. | AIDR | >999.999 | [ 700.93;>999.99] | <0.001 |  |  |
|  | |  |  |  |  |  |  |  |  |  |
|  |  |  |  |  |  |  |  |  |  |  |
| All statistics based on GEE. Patients and readers were included as random effects. Intercepts omitted for brevity; OR: Odds ratio; OR > 1: Method A is more likely to show a higher rating for given parameter compared to B; Adaptive Iterative Dose Reduction (AIDR); Deep-learning Reconstruction with lower dose (DL-1); Deep-learning Reconstruction with standard dose (DL-1-SD); Novel Deep-learning Reconstruction with lower dose (DL-2) | | | | | | | | | | |
